# A comprehensive sediment dynamics study of a major mud belt system on the inner shelf along an energetic coast

**DOI:** 10.1038/s41598-018-22696-w

**Published:** 2018-03-09

**Authors:** James T. Liu, Ray T. Hsu, Rick J. Yang, Ya Ping Wang, Hui Wu, Xiaoqin Du, Anchun Li, Steven C. Chien, Jay Lee, Shouye Yang, Jianrong Zhu, Chih-Chieh Su, Yi Chang, Chih-An Huh

**Affiliations:** 1 Department of Oceanography, National Sun Yat-sen University, Kaohsiung, Taiwan, ROC; 2 State Key Laboratory of Estuarine and Coastal Research, East China Normal University, Shanghai, China; 3 School of Ocean Science and Technology, Zhejiang Ocean University, Zhoushan, Zhejiang, China; 4 Institute of Oceanology, Chinese Academy of Science, Qingdao, China; 5 Taiwan Ocean Research Institute, National Applied Laboratories, Kaohsiung, Taiwan, ROC; 6 State Kay Laboratory of Marine Geology, Tongji University, Shanghai, China; 7 Institute of Oceanography, National Taiwan University, Taipei, Taiwan, ROC; 8 Department of Hydraulic and Ocean Engineering and Institute of Ocean Technology and Marine Affairs, National Cheng-Kung University, Tainan, Taiwan, ROC; 9 Institute of Earth Sciences, Academia Sinica, Taipei, Taiwan, ROC

## Abstract

Globally mud areas on continental shelves are conduits for the dispersal of fluvial-sourced sediment. We address fundamental issues in sediment dynamics focusing on how mud is retained on the seabed on shallow inner shelves and what are the sources of mud. Through a process-based comprehensive study that integrates dynamics, provenance, and sedimentology, here we show that the key mechanism to keep mud on the seabed is the water-column stratification that forms a dynamic barrier in the vertical that restricts the upward mixing of suspended sediment. We studied the 1000 km-long mud belt that extends from the mouth of the Changjiang (Yangtze) River along the coast of Zhejiang and Fujian Provinces of China and ends on the west coast of Taiwan. This mud belt system is dynamically attached to the fluvial sources, of which the Changjiang River is the primary source. Winter is the constructive phase when active deposition takes place of fine-grained sediment carried mainly by the Changjiang plume driven by Zhe-Min Coastal Currents southwestward along the coast.

## Introduction

Mud deposition systems are common features on continental shelves in the world^1,2^. They are active modern systems formed by complex interactions among single or multiple fluvial sources, physical processes, and the physiographic setting on multiple time and space scales. Most of these systems receive sediment from rivers and are considered as river-dominated sediment dispersal systems^2,3^. The shelf morphology and physical processes such as river plume, waves, and ocean currents that transport the fluvial sourced sediment determine the mud distribution pattern and the location of mud depocenters on continental shelves^1,4,5^. Since mud is very mobile, mud systems are easily subject to oceanographic processes, especially resuspension and transport by waves and currents. Consequently, on energetic shelves, constant mud deposition mostly occurs on the mid-shelf in deeper waters^6–8^. Mud-rich areas are also located in sheltered depressions^1^. In rare cases, when there is large supply of fine-grained sediment in low energy environments, mud deposits can be found on the inner shelf^5^.

There are eight types of mud deposition systems in the world based on the location of the depocenter on the shelf and their three-dimensional architecture. They include prodelta, subaqueous delta, mud patch, mud blanket, mud belt, shallow-water contourite drift, mud entrapment, and mud wedge^1^. Furthermore, these systems are actually conduits linking the source (at river mouths), processes (waves, tides), and sinks (shelf depocenters on margins). Based on this view, 5 types of fine-grained river-sediment dispersal systems have also been proposed: proximal-accumulation-dominated (PAD), marine-dispersal-dominated (MDD), estuarine-accumulation-dominated (EAD), subaqueous-delta-clinoform (SDC), and canyon-captured (CC)^2^. Although some of the types in the two classification schemes overlap, they nevertheless point out the diversity in these systems.

Since fine-grained sediments are carriers of geochemical signals and environmental proxies^9^, they preserve signals of river catchments and marine environments and thus the mud deposition systems also act as archives of paleoclimatic and paleo- and modern-environmental changes and coastal evolution during deglaciation^10–15^. Because of their archiving ability, they are also sinks for terrestrial substances and pollutants^16–18^, especially at depocenters.

These mud deposition systems reflect not only how the earth system functions at the land-sea boundary as influenced by the global change, they also reveal the nature of anthropogenic impact on the continental margin. Therefore, it is important to have a holistic understanding of the process and response that lead to the formation and maintenance of these systems.

**Figure 1:**
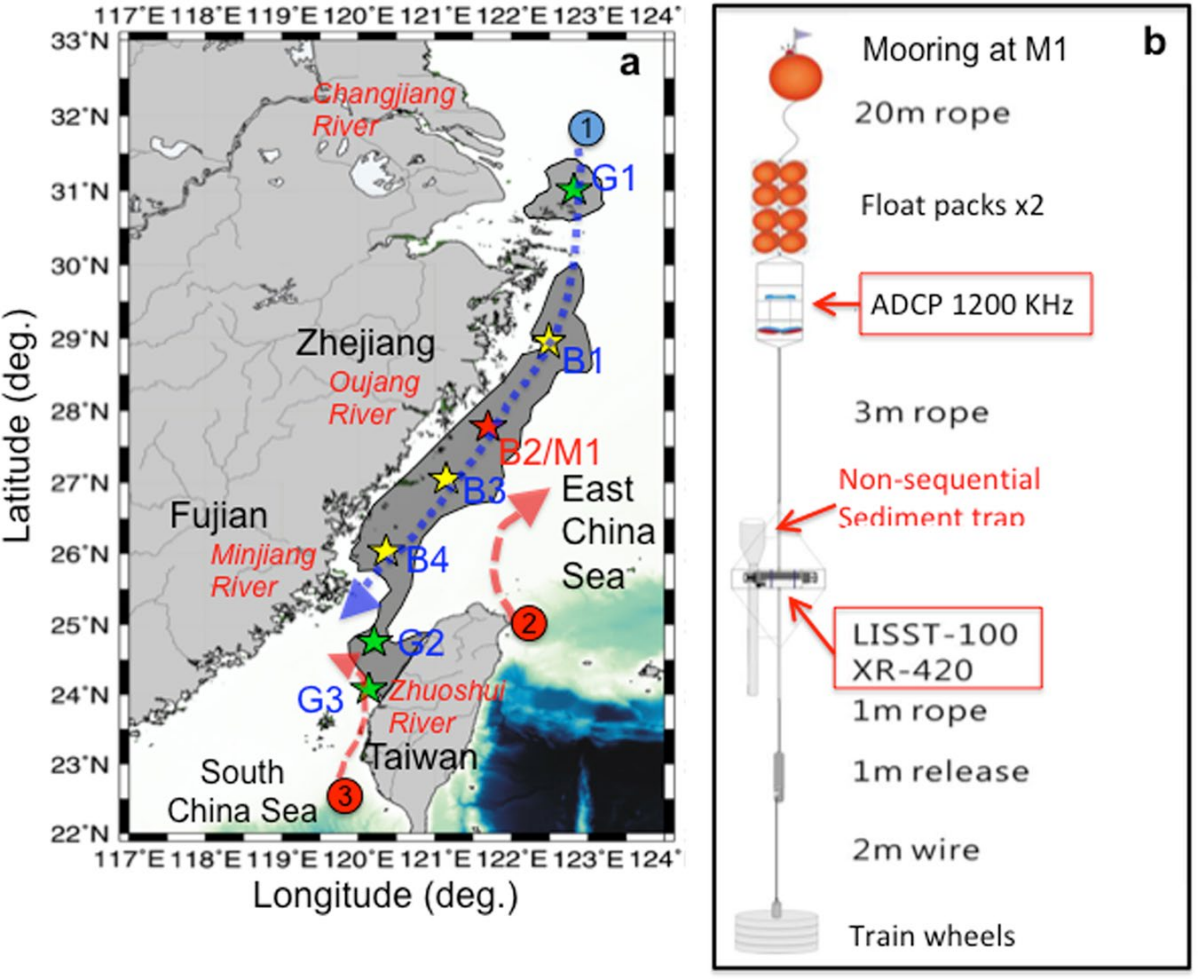
Map of the study area and the mooring configuration. (**a**) The region of the mud belt between the mouth of Changjiang River and the northern Taiwan Strait along the coast of Zhejiang and Fujiang Provinces of China. At each sampling station box core was taken. At Sta. G1, B4, and G2, gravity cores were taken successfully. Sta. M1 denotes the general location for the sediment trap mooring, wave/weather-station buoy, and the short shore-normal transect. The circled numbers marked regional current systems (shown as dashed arrows, blue for cold water, red for warm water) that affect the study area: 1. Zhe-Min Coastal Current, 2. Kuroshio Branch Current, and 3. South China Sea Warm Current. (**b**) Schematic plot of the sediment trap mooring that was configured with (in ascending order) an XR-420 (CTD with turbidity sensor) and LISST-100 × (Laser Particle Analyzer), a non-sequential sediment trap, and a downward looking ADCP (Aquadopp). All digitally acquired data presented have been synchronized on hourly intervals. The base map in (**a**) was plotted by GMT 5.1.2 software (http://gmt.soest.hawaii.edu) using the data from Taiwan’s Ocean Data Bank (http:/www.odb.ntu.edu.tw) with a grid resolution of 200 m. Details of the map including the silhouette of the mud belt were later added in PowerPoint.

In this study we focus on a major mud deposition system on the inner shelf of East China Sea (ECS) that extends from the mouth of the Changjiang (Yangtze) River along the coast of Zhejiang and Fujian (Zhe-Min in brief in Chinese) Provinces into the Taiwan Strait (TS) and eventually terminates on the west coast of Taiwan (Fig. 1a). The length of the system is about 1000 km having the width on the order of 100 km. The identity of this elongate mud region has not been clearly defined and is conventionally referred to as the mud zone, mud area, mud depocenter, mud wedge, and mud belt^2,11,13,14,19–23^. Although the northern terminus is clearly defined, the southern end has not been well defined because most studies have been done on the Mainland China side. In this study, we combined the schematic maps of the mud areas based on data from Mainland China^24^ and Taiwan^25,26^ to make a schematic composite map to delineate the entirety of the mud belt system (Fig. 1a).

The mud region along the Zhe-Min Coast has been well studied to show that this is an archive and sink for Changjiang-derived sediment and organic material^11,12,14,16,19,27–29^. Provenance studies of sediment along this mud belt based on clay minerals^19,23,28^, magnetic properties^30^, and radioactive nuclides^16^ indicate this mud belt is a conduit for both mainland and Taiwan-derived sediments, and thus, a source-to-sink system that links Chinese Mainland and Taiwan. Sub-bottom profile studies show that the thickness of clinoform and mud wedge decreases from the Changjiang submerged delta along the Zhe-Min Coast^22,23,27,28,31^.

Dynamically this system is subject to the strong monsoonal seasonality of the river discharge and related plume dynamics, coastal currents in the ECS such as the Zhe-Min Coastal Current (ZMCC) and the intrusion of Kuroshio Branch Current (KBC), South China Sea Warm Current (SCSWC), and wind and wave fields^32^. The energetic tidal system in the ECS also affects the dynamics. Many of these processes have been suggested to disperse Changjiang-derived fresh water and sediments and transport southward along the mud belt, such as the Changjiang Diluted Water (CDW) that forms a narrow band attaching to the Zhe-Min Coast^33^ by the ZMCC^21,34^. Additionally, the buoyant CDW and the more saline KBC water create haloclines^35^ on the ECS shelf, forming stratification that affects the vertical structure of suspended sediment, which in turn, affects sediment transport along the mud belt^21,34^. Therefore, this is quite a complicated sediment dispersal system.

Despite the complexity in the dynamics of the system, there have not been studies that directly link the dynamics of sediment to the dispersal of the fluvial source, sediment settling and transport, and eventual deposition along this conduit. Fundamental questions regarding the dynamic maintenance of this system have yet to be answered with certainty. Specifically, how is the mobile mud retained on this energetic shallow inner shelf? What prevents the mud to get dispersed to deeper waters as it does in other systems? How does the river-derived sediment enter the system? What is the evidence in the settling particles in the water column to link the fluvial sources to the seafloor sediment? In order to the address these questions, we designed a comprehensive process-based study. We used an instrumented mooring with a sediment trap to investigate the coupling between the temporal changes in the flow field to water temperature, and salinity and suspended sediment concentration over two spring-neap cycles. We used using shipboard observations to investigate the coupling between structures in the water column (salinity, temperature, density, and chlorophyll-a) and suspended sediment distribution, to establish cause-and-effective relationship over a diurnal tidal cycle. We compared the properties of settling particles captured in the sediment trap to hypothetical fluvial sources and establish direct provenance of settling particles. Additionally we examined the evidence from the seafloor surficial sediment and sediment cores along the mud belt to establish source-to-sink characteristics of the mud belt. Our goal is to integrate all the measurements of the water column and the corresponding suspended and seafloor sediments to establish a process-based model of this dispersal system that links the mouth of Changjiang River and the west coast of Taiwan.

In our field campaign R/V Ocean Researcher 5 (OR-5) deployed a sediment trap mooring off the mouth of Oujiang River on Feb. 17, 2014; took seafloor sediment samples, and box and gravity cores along the entire mud belt (Fig. 1b). A chartered fishing boat also deployed a wave/weather station buoy near the mooring site and conducted hydrographic profiling along a shore-normal transect nearby. On March 17 R/V Ocean Researcher 1 (OR-1) conducted a 24-hour hydrographic profiling and took water samples at 3 m depths near the mooring before retrieving it.

## Results

### Influence of the weather, tides, the Changjiang buoyant plume, and ZMCC

**Figure 2:**
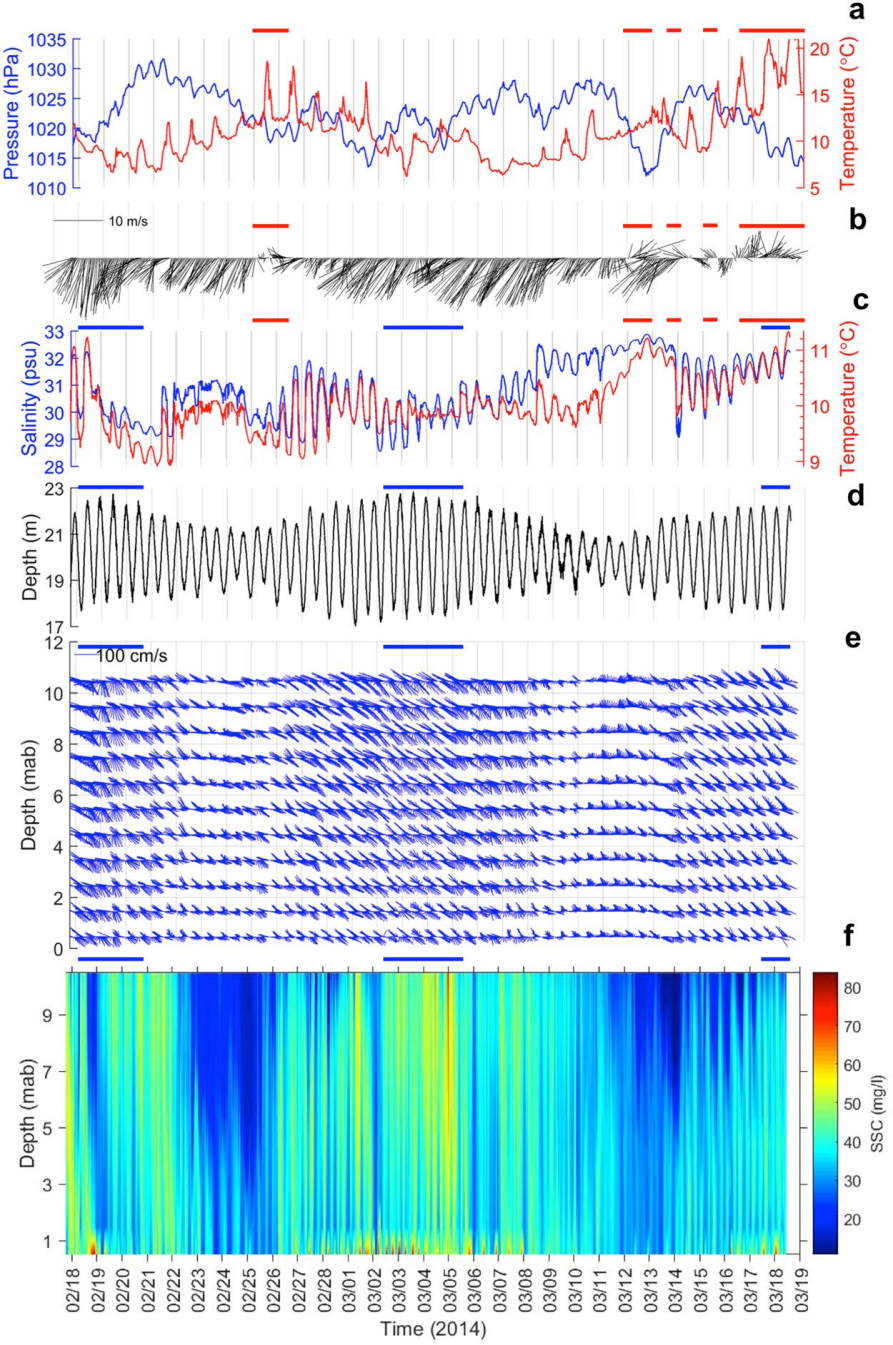
Measured time series from Feb. 18 to March 19. Meteorological variables measured by the wave/weather station buoy included (**a**) atmospheric pressure and temperature; and (**b**) wind speed and direction (stick diagram plotted according to the oceanographic convention, the upward direction is north). Hydrographic variables by the mooring included (**c**) salinity and temperature and (**d**) water depth variability from XR-420; (**e**) speed and direction of the instantaneous flow from the ADCP at 1 − m intervals (as stick diagram, upward direction is north; and (**f**) converted suspended sediment concentration (SSC) from the echo intensity of the ADCP at 0.25 m intervals. The red horizontal bars at the top of (**a**,**b**,**c**) indicate periods of southerly winds. The blue horizontal bars at the top of (**d**,**d**,**e**,**f**) indicate periods of spring tide.

During the deployment, the winter monsoon climate dominated the study site as shown by the high-pressure atmospheric system associated with cold air temperature (Fig. 2a) and strong NE winds (Fig. 2b). However, bursts of low atmospheric pressure, warm air temperature, and southerly winds punctuated the study period. In the fluctuating signals of salinity (33~28 psu) and temperature (9~11 °C) measured on the mooring the CDW^33^ water was evident as shown by the close simultaneous associations of low salinity and temperature (Fig. 2c). This association was stronger during spring tide than neap tide (Fig. 2d) since the tide exerted the first order influence on the flow^36,37^ measured in the lower water column between the seabed and 11 m above the bed (Fig. 2e). Therefore, the flow was stronger in spring tide, and weaker in neap. Closer to the seabed, the flow became weaker. Temporal changes of the flow field in the vertical are better seen in the progressive vector plots at 1-m intervals (Supplemental Fig. S1). The net direction of the flow between 5–10 mab was SW parallel to the coast, which was driven by the ZMCC^37,38^. Below 5 mab, the net flow direction was NE along the coast, which was influenced by the intrusion of KBC^39–42^. Consequently, a horizontal shear front located at 5 mab could exist, which might hinder the vertical exchange of SSC. This issue will be addressed later.

Water movement of individual tidal cycles was mainly in the on-offshore direction. The tidal influence is also reflected in the temporal changes in suspended sediment concentration (SSC) profile in the lower part of the water column (Fig. 2f). Higher values corresponded to the time of spring tide both near the seabed and above the seabed. Temporal changes in the cumulative sediment transport in the lower water column had patterns similar to those of the water movement (Supplemental Fig. S2). Net suspended sediment movement was southwestward along the coast in the upper part of measured water column and northeastward near the bottom with reduced magnitude. Further quantification on the tidal influence on the temporal changes of salinity, temperature, flow, and wave field is presented in Supplemental Information. Basically, except for the on-offshore flow component and water depth, tide is not a major forcing in water-born variables in the water column on the time scale of a month (Supplemental Table S1).

### Influence of the KBC intrusion

**Figure 3:**
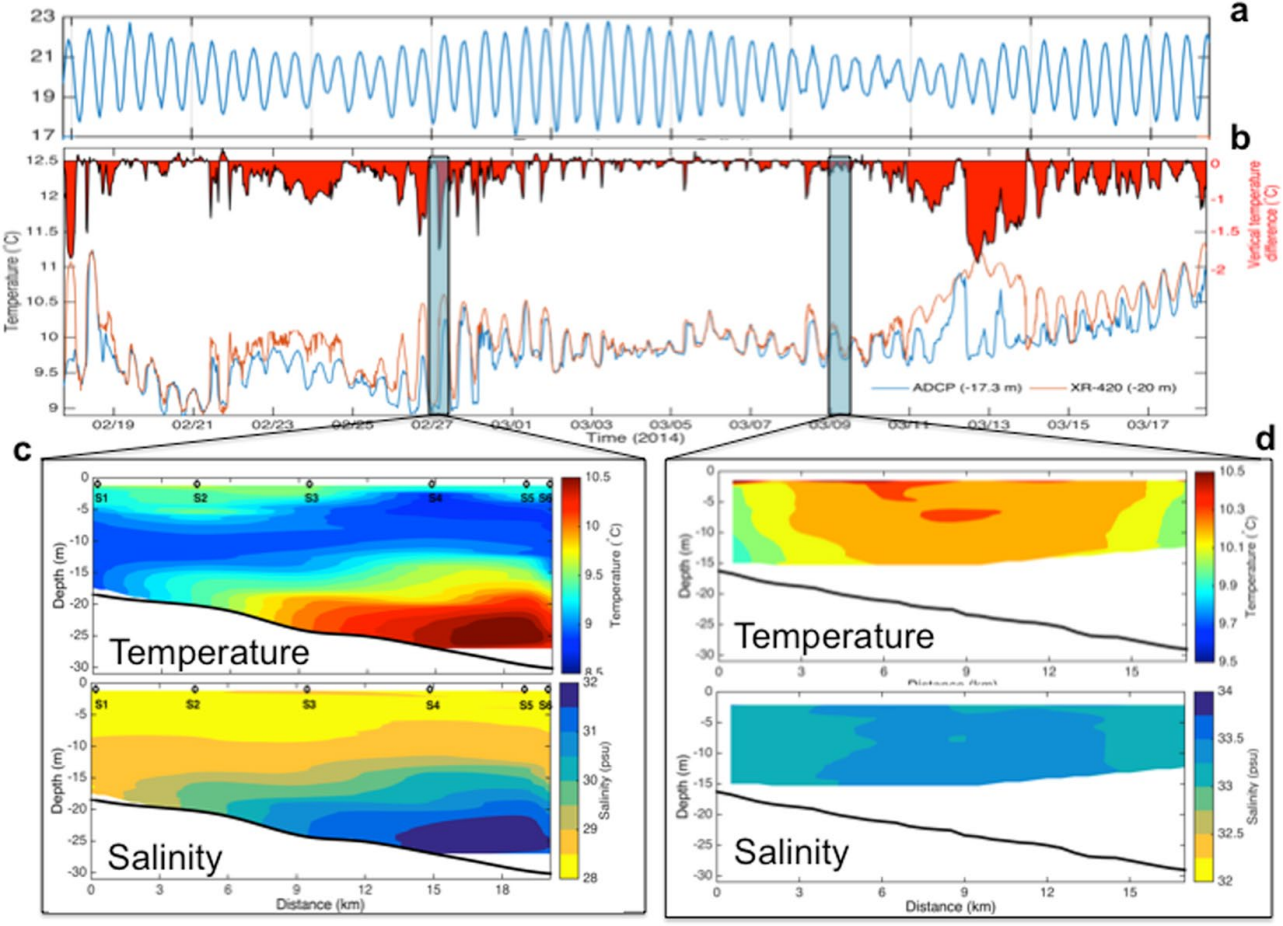
Water column stratification and de-stratification. (**a**) Water depth fluctuations are shown to indicate tidal stages (**b**) Temperature difference (upper minus lower, negative values indicate lower water column was warmer) between depths at −17.3 (measured by ADCP) and −20 mab (measured by XR-420) Contour plots of the temperature and salinity along the shore-normal transect (Fig. 7a) on Feb. 27 (**c**) and March 9 (**d**).

KBC intrusion brought warmer but more saline Kuroshio subsurface water^39,40^ to the study site as seen in the warmer temperature at −20 mab than that at −17.3 mab measured on the mooring, resulting in sharp temperature reversals (warmer water beneath colder water, Fig. 3b), which was co-influenced by tide and wind. In spring tide, little temperature difference existed (Fig. 3a,b). Favorable conditions for the intrusion occurred when the wind was from the south during the neap (Figs 2b,d and 3b). Alternatively the enhanced temperature reversal during neap could be caused by reduced tidal mixing. The KBC intrusion can be also visualized by the temperature and salinity structures along a short shore-normal transect near Sta. M1 taken on Feb. 27 (Fig. 3c) and March 9 (Fig. 3d). On Feb. 27, strong stratification (Fig. 3b,c) coincided with the end of southerly winds (Fig. 2b), which could have enhanced the intrusion of KBC subsurface water. On March 9, the wind was from the NE, but the Changjiang discharge was likely weakened as indicated by increased salinity at the mooring site (Fig. 2c), so the KBC subsurface water reached the surface (Fig. 3d), forming an outcrop of saline and warm water in the cold and less saline coastal water (Fig. 3d).

### Water column structures over a diurnal tidal cycle

**Figure 4:**
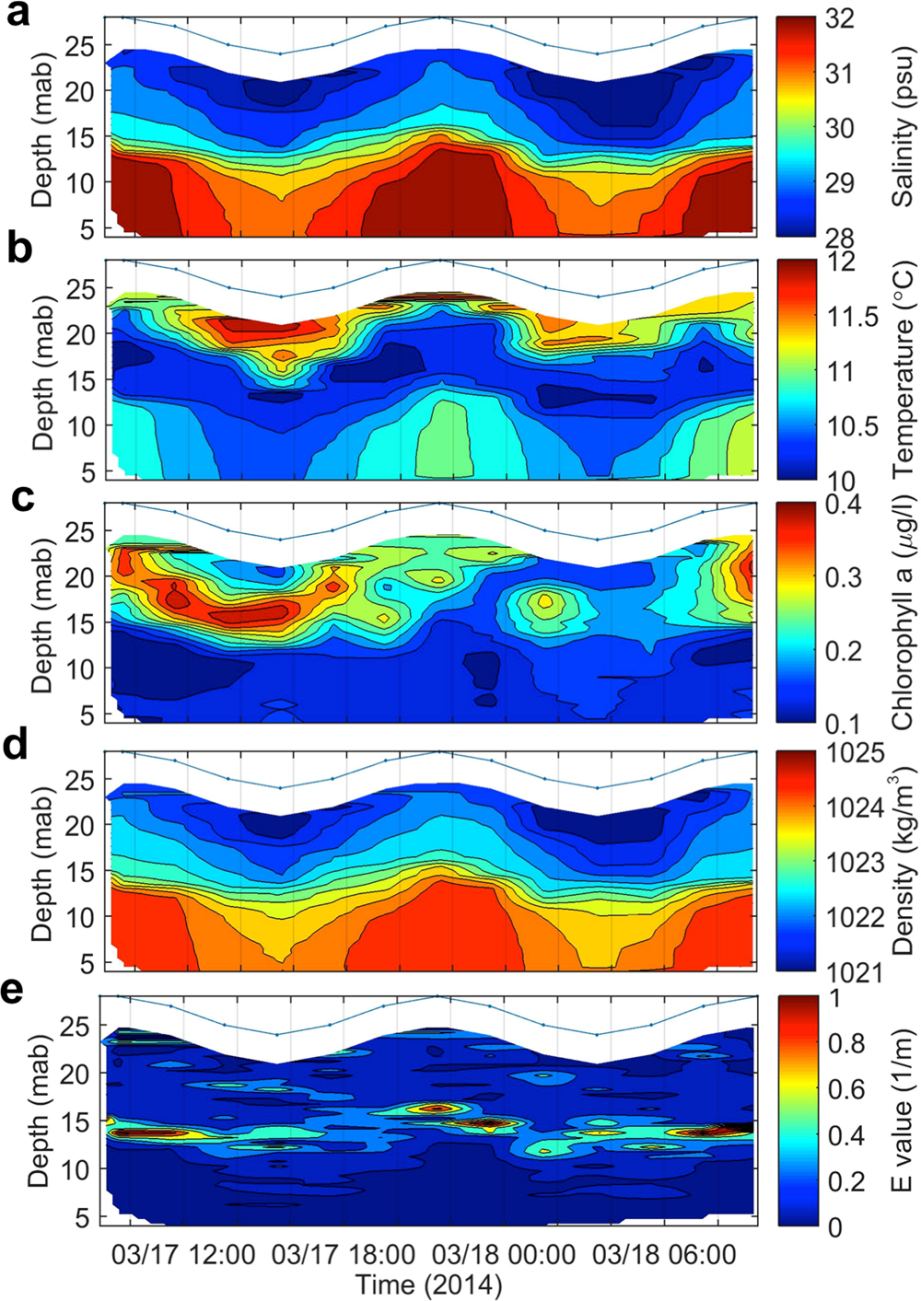
Water column variability over a diurnal tidal cycle. Structures of (**a**) salinity; (**b**) temperature; (**c**) chlorophyll-a; (**d**) water density; and (**e**) Static Stability (E) over a diurnal tidal cycle at Sta. M1 from March 17–18.

**Figure 5:**
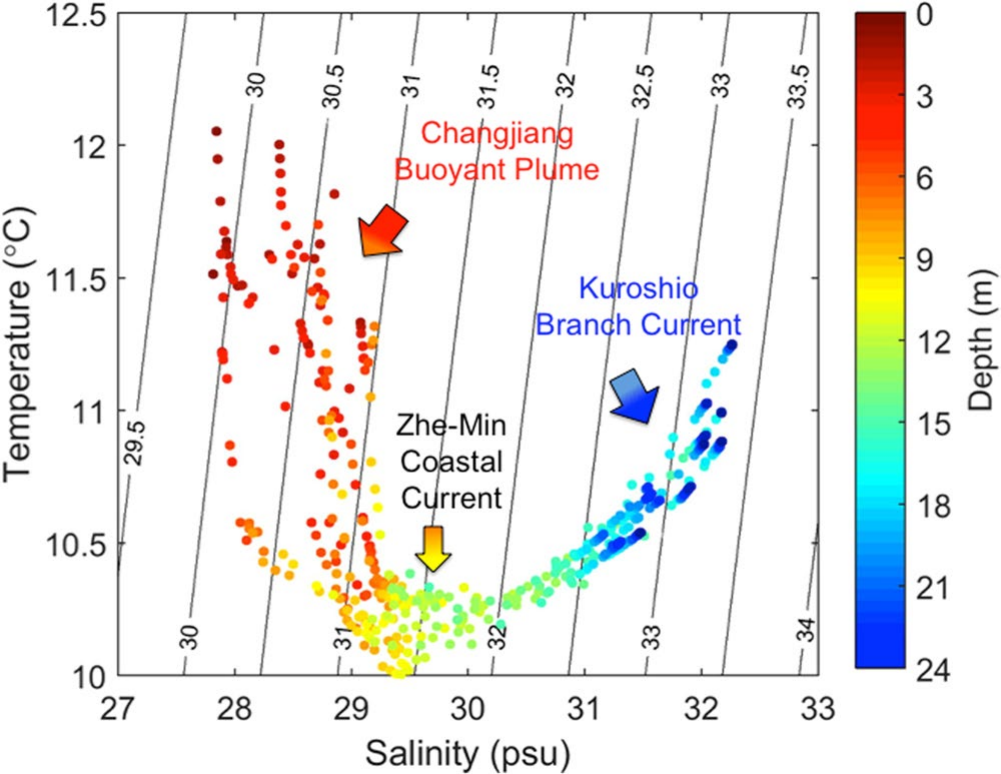
Depth-referenced T-S diagram. Located at different water depths, three water masses are identified, the Changjiang buoyant plume water in the upper water column, Zhe-Min Coastal Current water at mid-depth, and Kuroshio Branch Current water in the lower water column.

The vertical structures of salinity, temperature, chlorophyll-a, water density, and Static Stability (E) showed pronounced changes over a diurnal tidal cycle on March 17–18 at Sta. M1 (Fig. 4). During the ebb (from high water to low water) the upper water column contained less saline but warmer water with high chlorophyll-a (Fig. 4a–c) of the Changjiang buoyant plume, which became the thickest at low water. During the flood, the upper layer of buoyant plume became thinner. In the lower water column, the flood brought more saline but warmer subsurface water with low chlorophyll-a, which could be KBC water. Fresher plume water in the surface layer and saline oceanic subsurface water forming temperature inversion has been widely observed off the Chinese coast^35^. However, a thin layer of the coldest and less saline water existed between the tidally influenced layers of upper and lower water column has never been reported. This layer is also reflected in the density structure (Fig. 4d), which was the ZMCC water^37^. Consequently, a stable thin layer existed at mid-depth as indicated by the high E values (Fig. 4e), whose significance will be elaborated on in later sections. It should be noted that the max. E values coexisted with high tide, suggesting the flood tide in the shore-normal direction could enhance the KBC intrusion. The sandwich structure in the density field of the water column is due to the three water masses that occupied different depths as seen in the depth-referenced T-S diagram (Fig. 5). The Changjiang buoyant plume water, being the least saline, was located in the upper water column. The ZMCC water, being the coldest, was located at mid-depth. The KBC water, being the most saline, was located in the lower water column.

### Resuspension of seafloor sediment and sediment transport

**Figure 6:**
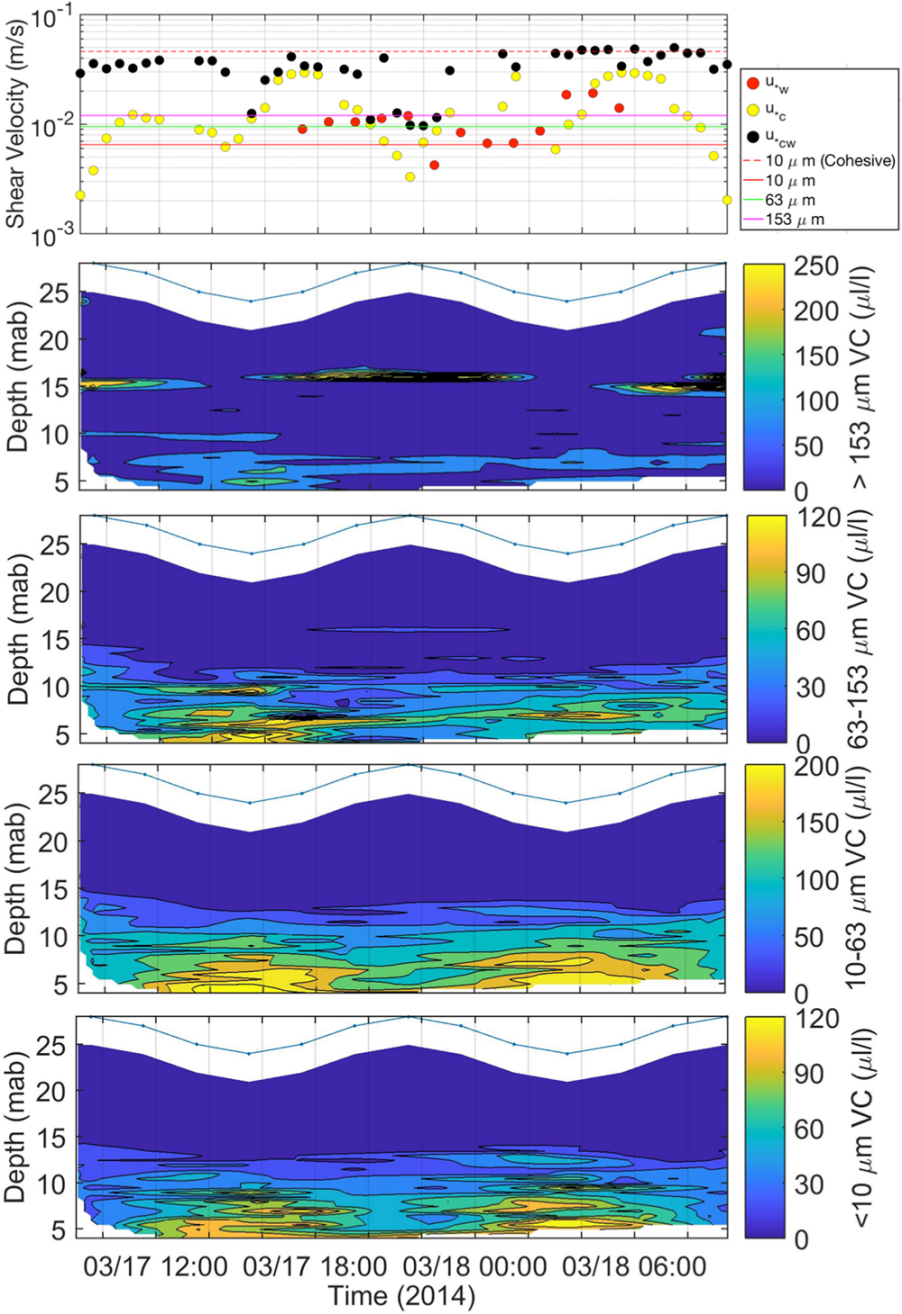
Entrainment of seafloor sediment. (**a**) Entrainment thresholds of three grain sizes of 10, 63, and 153 μm of non-cohesive sediment (horizontal lines) and one grain size of 10 μm of cohesive sediment^60^ (dashed horizontal line) and shear stresses (circles) induced by waves (U_*w_), currents (U_*c_), and wave-current interaction (U_*cw_); Temporal variability of the distribution of volume concentration in the water column of grain sizes (**b**) >153 μm; (**c**) 63–153 μm; (**d**) 10–63 μm; and (**e**) <10 μm from March 17–18 at Sta. M1.

The shear stresses (U_*_) induced by waves, currents, and wave-current interaction over a diurnal tidal cycle were estimated using the method described before^43,44^ were compared against entrainment thresholds of non-cohesive sediment of the grain sizes of 10, 63, and 153 μm, respectively (Fig. 6a). Since the mud on the seafloor was likely cohesive, thus the threshold for cohesive sediment of 10 μm was also plotted (Fig. 6a). The results show that the shear stress induced by waver-current interaction (U_*cw_) was the most important mechanism to resuspend seabed sediments of all three non-cohesive size classes. Furthermore, U_*cw_ barely reached the entrainment threshold for the cohesive sediment of 10 μm size during the flooding tide beginning at 06:00, March 18. Therefore, fine-grained cohesive sediment could be resuspended as well. The volume concentrations of grain-size classes of 63–153, 10–63, and <10 μm only appeared in the lower water column, having two cores of high values corresponding to low tides (Fig. 6c–e). These patterns suggest a resuspension mechanism that corroborates with Fig. 6a. However, the coarsest size of >153 μm showed weak resuspension in the lower water column, some presence in the upper water column and a strong presence as a thin layer that co-located with high E values at mid-depth (Figs 4e and 6b). This points to a set of similar mechanisms as the result of coupling between physical processes (mixing vs. stratification) and large-sized biogenic particles such as phytoplankton that contains chlorophyll-a observed off the mouth of Minjiang River^44^ (Fig. 4c). The average total SSC (mg/l) measured from water samples taken onboard OR-1 was 46.9 mg/l at the surface, 46.2 mg/l at mid-depth, and 298.5 mg/l near the seabed. These values corroborate with the volume concentration measurements that there were more suspended sediments in the lower water column. At all three depths, the suspended sediment consisted mostly (over 80% by weight) of clay-sized particles (Supplemental Fig. S4).

### The significance of a mid-depth dynamic barrier in sediment transport

**Figure 7:**
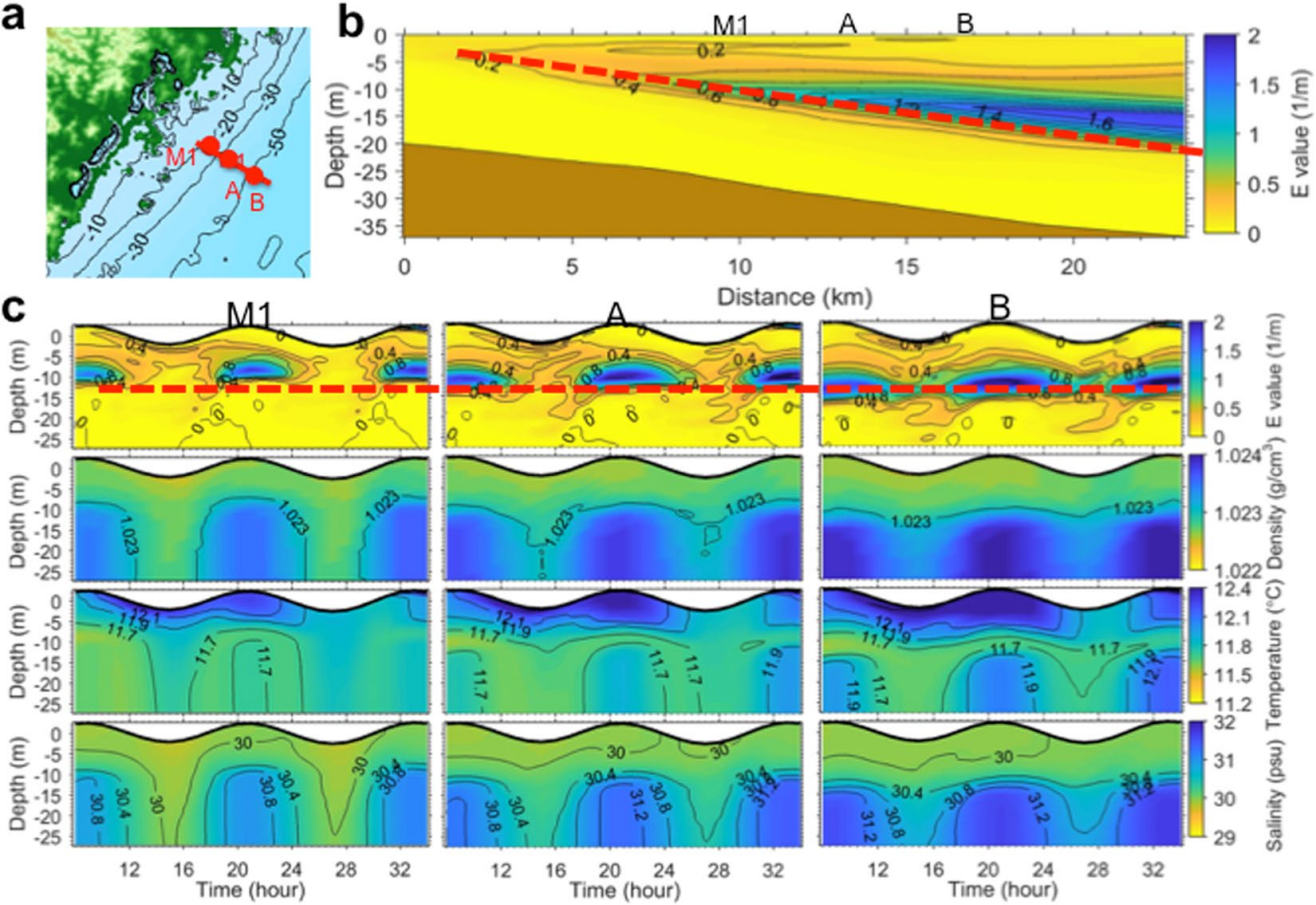
Numerical simulations of water-column hydrodynamics. (**a**) Map showing the location of the short transect along which results are displayed. (**b**) Simulated structure of the Static Stability (E) along the transect showing checkpoints at M1, A, and B. (**c**) Simulated diurnal tidal cycle of the water-column structure at M1, A, and B of (from top to bottom) Static Stability (E); density; temperature; and salinity. The red dashed line indicates the dynamic barrier created by E. The map in (**a**) was plotted by MATLAB 8.0b software (http://gmt.soest.hawaii.edu) using the data from Taiwan’s Ocean Data Bank (http:/www.odb.ntu.edu.tw) with a grid resolution of 200 m. The short transect and locations of M1, A, B were later added in PowerPoint.

The structures of suspended sediment in the water column and the corresponding presence of the stable layer at mid-depth suggest a mechanism that has a great significance on the sediment dynamics of the mud-belt transport system. Therefore, it is important to verify the layer of high E and its spatial and temporal characteristics. Subsequently a numerical model was used^36,38^ to simulate the observed water column structure at Sta. M1 and along a short shore-normal transect (Supplemental Information). The results show that a layer of high E values at mid-depth could be simulated along a shore-normal transect (Fig. 7a,b). At Sta. M1 and two other checkpoints A, B, the temporal and spatial changes of E were sufficiently reproduced over a diurnal tidal cycle (Figs 4e and 7c).

**Figure 8:**
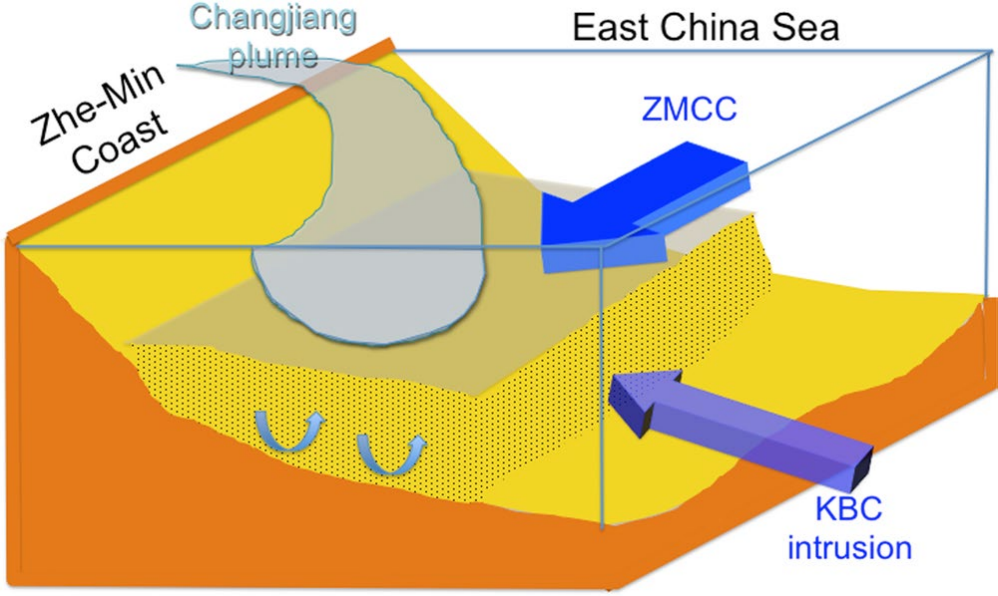
Schematic drawing to show the mid-depth barrier created by the three water masses that inhibits upward mixing of suspended sediment. The less saline Changjiang buoyant plume in the upper water column, the coldest Zhe-Min Coastal Current at mid-depth, and the most saline Kuroshio branch water in the lower water column jointly create a strong horizontal stratification front at mid-depth that restricts sediment resuspended by wave-current interaction to be further mixed upward and dispersed and transported in the upper water column.

Consequently, a conceptual model for critical physical processes that affect sediment dynamics of the mud belt along Zhe-Min Coast is presented in a schematic drawing (Fig. 8). The stratification related to the interplay among the Changjiang buoyant plume, ZMCC, and the KBC intrusion formed a horizontal dynamic barrier at mid-depth. Although the near-bed shear stresses induced by current-wave interaction are sufficient to resuspend non-cohesive sediment up to fine sand (153 μm) and fine-grained cohesive sediment from the seabed into the water column, the dynamic barrier prevents the suspended sediment to be further mixed upward. Consequently, the resuspended sediment remains in the lower water column close to the seabed. A flow front could be caused by the density stratification, which capped the bottom Ekman layer and weakened the upward mixing of resuspended sediments. Therefore, suspended sediment could only be transported by weakened currents in the lower part of the water column.

Although the concept of stratification has been proposed to influence sediment transport for summer^21^, our findings are the first to quantify this mechanism for the winter season when the sediment transport is the strongest^21^. The low SSC values measured in the upper water column was mainly due to the fine-grained sediment carried by the Changjiang buoyant plume including a small fraction of larger biogenic particles (Supplemental Fig. S4). These particles can still settle to the bottom through the dynamic barrier due to their own density, which is much higher than that of the seawater.

### Sinking particles were sourced from the Changjiang River, Oujiang River, and Zhuoshui River

**Figure 9:**
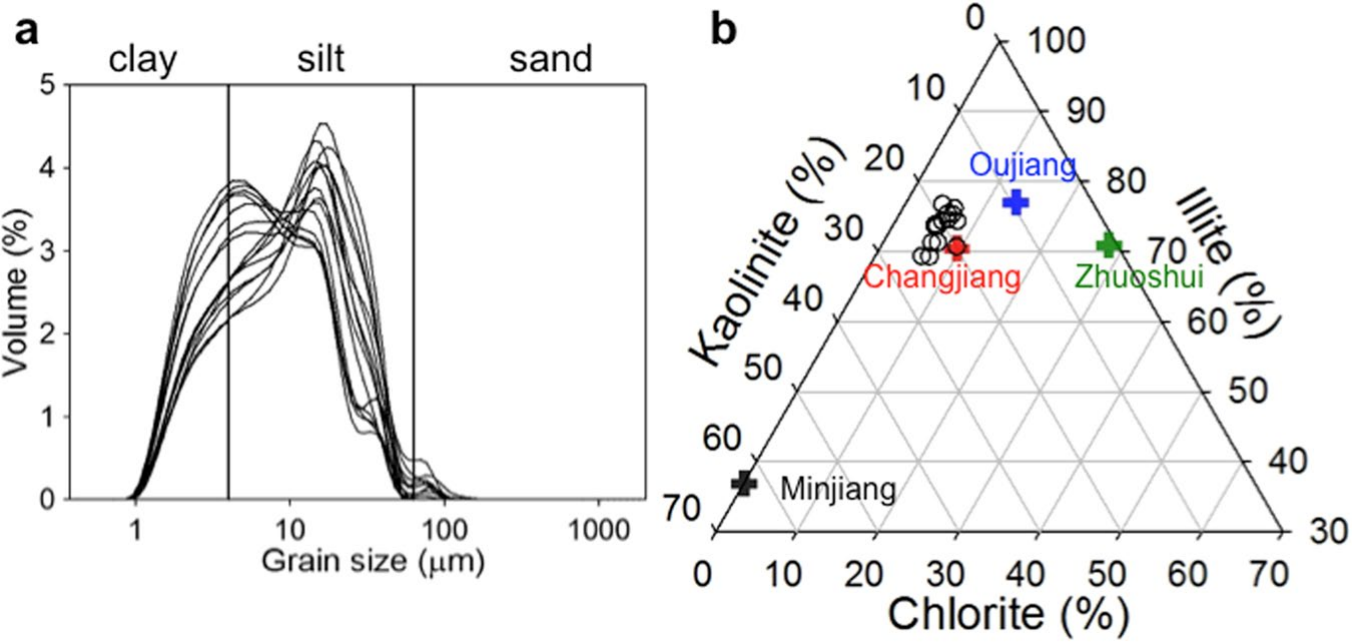
The texture and sources of the sediment captured in the sediment trap. (**a**) Grain-size distribution of sediment trap samples. (**b**) Tertiary plot of the three representative clay minerals showing differential affinity of the sediment trap samples to four hypothesized fluvial sources of Changjiang River, Oujiang River, Minjiang River, and Zhuoshui River. Note: the fluvial sources were based on (ref.^28^).

Numerous multiproxy studies on sediment cores and seabed samples^12,18,23,27,28,42^ suggested that the Changjiang River is the source for mud belt along Zhe-Min Coast. Analyses of samples taken from the 15-cm thick sediment accumulated in the sediment trap, we obtained for the first time characteristics of sinking particles (Supplemental Information) above the mud belt. These particles were fine-grained, predominantly in clay and silt fractions (Fig. 9a). Content of three major clay minerals, kaolinite, illite, and chlorite, in the sediment trap were compared to representative values of those in hypothetical fluvial sources, the Changjiang River, Oujiang River, and Mingjiang River along Zhe-Min Coast, and the Zhuoshui River across the TS. The result showed the sediment trap samples had the highest affinity to Changjiang River, then to Oujiang River, and lastly to the Zhuoshui River. Minjiang River was unlikely to contribute clay minerals to the sediment trap samples (Fig. 9b). Additional information was extracted from co-varying properties of sediment trap samples including the percentages of clay, silt, and sand, total ^210^Pb, TN, TOC, F_t_ (terrestrial fraction), illite, kaolinite, chlorite, quartz, k-feldspar, plagioclase, and calcite (Supplemental Fig. S6), using Empirical Orthogonal/Eigen Function technique (Supplemental Information). The result showed the most important mode of co-variability (43.4%) among sample properties was the separation of clay-sized particles form coarser particles due to hydraulic sorting in the sediment transport process. In this mode terrestrial signals positively co-vary with clay-sized particles (Supplemental Fig. S7). Secondary (15.8%) and tertiary (14.2%) modes of co-variability were caused by the provenance contrast between weathered granitic sources from rivers in Zhe-Min Provinces vs. Changjiang source; and coarse clastic (nearshore/marine sourced) particles vs. silty organic material (terrestrial sourced), respectively (Supplemental Fig. S7).

### Changjiang River and the Zhuoshui River are the primary and secondary sources for fresh terrestrial sediments

**Figure 10:**
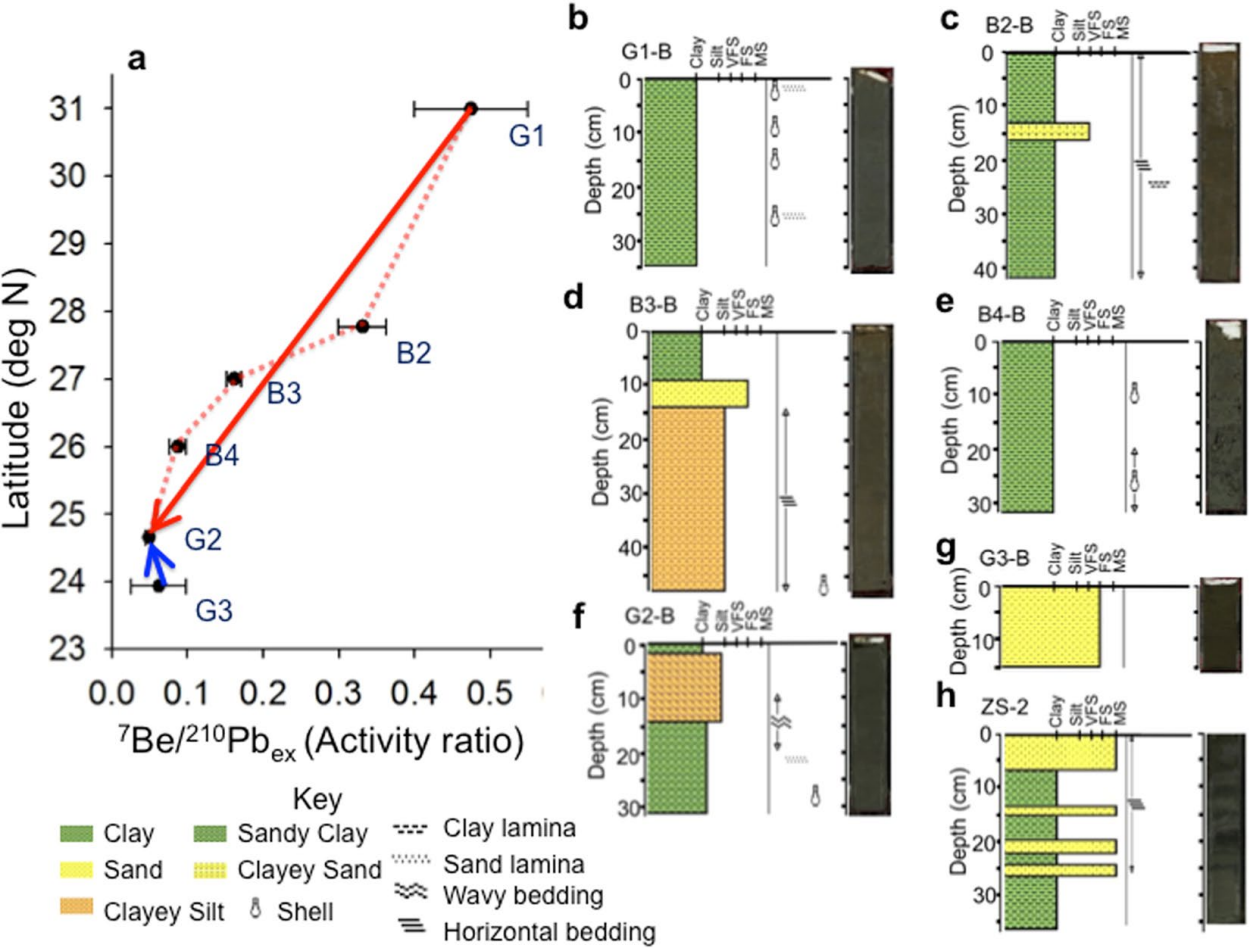
Sea-floor evidence showing the sources of the mud belt. (**a**) Spatial variability of ^7^Be/^210^Pb_ex_ along the mud belt between the mouth of Changjiang River at G1 and northern Taiwan Strait at G2. The red arrow indicates inferred transport from G1 to G2 and the blue arrow indicated inferred transport from G3 to G2. Lithology, internal structure, and photo of each box core taken in this study at Sta. (**b**) G1; (**c**) B2; (**d**) B3; (**e**) B4; (**f**) G2; and (**g**) G3. (**h**) A box core (ZS-2) taken by another study in summer 2015 about 17 km away from Sta. G3. Abbreviated symbols represent very fine sand (VFS); fine sand (FS); and medium sand (MS).

Evidence from the seabed provided additional indication on the sources and deposition along the mud belt. A decreasing trend of ^7^Be/^210^Pb_ex_ values from G1 to G2. (Fig. 1a) suggests the decrease in the freshness of terrestrial sediment along the mud belt (Fig. 10a). However, another decreasing trend also exits from G3 to G2, suggesting contribution from Taiwan. These trends indicate that on the time scale of 200 days, fresh terrestrial sediment was transported SW from the mouth of Changjiang River along the mud belt to northern TS and from Taiwan along the TS mud belt in the NW direction. The lithology and photos of box cores taken from the substrate at Sta. G1, B2, B3, and B4 along Zhe-Min Coast, and at G2 on the TS mud belt show clay, to sandy clay, and clayey silt deposits with occasional shell fragments, and some physical structure of clay and sand lamina, and horizontal and wavy beddings (Fig. 10b–f). In Cores B2 and B3 single layers of clayey sand and sand a few centimeters thick are present, respectively. Since G3 was located on the moribund sand ridge of the Changyun Ridge (CYR)^45^ which is outside the boundary of the TS mud belt (Supplemental Fig. S8), the entire box core is composed of sand (Fig. 10g). For comparison, another box core ZS-2 taken about 17 km away inside the TS mud belt in summer (Supplemental Information) is composed of interlayered sand and clay (Fig. 10h). The alternate sand and mud deposits in this core suggest the influence of the dual sources of the CYR and Zhuoshui River nearby. The three longer gravity cores at G1, B4, and G2 showed decreasing thickness of clay from the mouth of Changjiang River to the mud belt in the TS (Supplemental Fig. S9). The overall attributes of box and gravity cores suggest that the fine-grained (clay size) sedimentary process was the strongest at the northern end of the system and the physical processes dominated the sedimentation. At the mid-point of B4, biological influence became important part in the sedimentary process and the supply of Changjiang-sourced clay decreased. At G2, which is at the junction between the Zhe-Min mud belt and the TS mud belt (Supplemental Fig. S8a), the dominant sedimentary features were silt deposition in which physical and biological processes were both important. The thinning of the mud deposit is also corroborated by the pinching-out of Changjiang-derived clinoform in the northern TS^22^.

## Discussion

Our findings suggest that the mud area shown in Fig. 1a is an active sediment dispersal system, whose major fluvial source is the Changjiang River at the northern terminus, and the Zhuoshui River at the southern terminus is a secondary source. Since the textural expression of this system on the seafloor is mud (clay and silt), we propose to call it Zhe-Min-Taiwan Strait Mud Belt (Z-M-TSMD). Contrasting to the existing description of mud belt systems^1^ the Z-M-TSMD is the result of semi-continuous delivery of fresh and clastic fine-grained terrestrial sediment from the two major fluvial sources at both termini in winter. There are also other minor sources from rivers that drain the granitic terrain of the Zhe-Min Provinces such as the Oujiang River. Additionally, marine-sourced particles also contribute to the sinking particles over the mud belt. The ZMCC is the primary forcing to cause the Changjiang buoyant plume to have the shore-hugging path along the Zhe-Min Coast. Another branch of the Kuroshio that enters the TS through the Luzon Strait flows northward on the east side of the TS. This current is impeded by the shallow CYR in the middle section of the TS and is diverted northwestward^46^ (Fig. 1a). This could contribute to the northwestward transport of Taiwan-sourced sediment, thereby extending the mud belt from Taiwan to join the mud belt on the Zhe-Min Coast. In this system, there is close coupling between the flow field and suspended sediment field. There is also close coupling between the settling mud carried by the Changjiang plume water and the mud on the surface of the seafloor and between deposits in the substrate. Therefore, the Z-M-TSMD is closely attached to the sources, not detached as described before^1^. We also see homogeneous mud deposit (Fig. 10b–g, Supplemental Fig. S9a,b), although thinning from the primary source without a noticeable fining upward trend, which is different from previous description^1^. Our findings do allow us to evaluate the mixing or unmixing of fines from the margins toward the deposition center at the southern terminus based on the two adjacent cores G3 and ZS-2 (Supplemental Fig. S8b). Since CYR is predominately sand, the mud seen in Core ZS-2 (Fig. 10h) had to come from the Zhuoshui River. Furthermore, based on previous studies that fine-grained sediment only exits on margins along the Zhe-Min and Taiwan coasts^22,23,28,31^, it is unlikely that the inner shelf mud would come from offshore or relict sediment in the center of Taiwan Strait^25,47^. In the maintenance of the Z-M-TSMD, it is crucial that fluvial sourced mud (clay and silt) is constantly supplied in winter.

The retention of the mud settled to the seafloor is also very crucial in the maintenance of the system. Our findings point to the stratification-related horizontal dynamic barrier (defined by high E values) as the key mechanism under winter conditions. This barrier restricts suspend sediment to the lower water column where the suspended concentration is high. Yet the weaken flow in the lower water column limits effective sediment transport. This barrier in the vertical is likely to exist in the confines of the Changjiang buoyant plume or CDW transported by ZMCC. Furthermore, the three-D density structures, due to the Changjiang buoyant plume and KBC, create shore-parallel vertical isopycnal surfaces^48^ that could act as lateral barriers to prevent suspended mud to escape seaward, further confining the mud within the mud belt. In winter the expression of the vertical isopycnal interface at the sea surface can be seen in satellite-derived sea surface temperature gradients, which form a frontal zone that corresponds well with the seaward boundary of the mud belt along Zhe-Min Coast (Supplemental Fig. S10). This front also forms a bend in the northern TS whose apex corresponds to the region where Taiwan-derived mud belt joins the mud belt along the Zhe-Min Coast (Supplemental Fig. S9a). Therefore, it is clear that the lateral extent of the Z-M-TSMS is defined by the frontal region between ZMCC and KBC and the ambient waters of ECS and TS.

However, our data did not cover the summer season, we therefore speculate based on known facts. In summer the dispersal of CDW is in the NW direction, it does not overlap with Z-M-TSMS. Yet, summer is the flood season of all rivers in the region, the effluent from other rivers along the mud belt replaces the role of the Changjiang buoyant plume and the vertical stratification in the water column still exists^21^. A sediment dynamics study off the mouth of Minjiang River in summer shows under contrasting wind conditions of SW summer monsoon and NE typhoon winds, a stratified/isopycnal layer existed at mid-depth^44^. Under both conditions a benthic nepheloid layer below the dynamic barrier formed by the isopycnal layer was also present. Thus, the ‘entrapment mechanism’ to prevent upward mixing of suspended sediment near the sea floor still exists in summer. Since ZMCC is weakened or replaced by northward flowing SCSWC, Changjiang River would not be a major source for fluvial sediment and the predominant sediment transport direction is northward^21^. Therefore, the Z-M-TSMD is likely to have two phases in the course of a year. During the winter monsoon season, the system receives ample fluvial sediment from the Changjiang River via southward sediment transport, and the deposition on the sea floor is active and the vertical and lateral dynamic barriers are well in place. This is the constructive phase. When the summer monsoon dominates, the system receives sediment delivered from small rivers on the Zhe-Min Coast, but little from Taiwan^49^. The buoyant water and colder and more saline water brought to the coast by upwelling forms stratification that could create a similar barrier in the vertical^21,32,44^. The amount of suspended sediment in the lower water column is less than that in winter^34^. Episodic typhoon events are likely to interrupt sediment transport and cause non-deposition^21^. They also deliver a large amount of fluvial sediment to the system. The impact of the typhoon on the system is yet to be evaluated. Nevertheless, the system is likely in a maintenance or non-constructive phase. The two-phase scenario fits the description of oceanic fronts and hydrodynamic and suspended sediment transport in this region^34,50^. Overall, the Z-M-TSMD is directly attached to the fluvial sources and is proximal-accumulation-dominated with a decreasing degree from the major fluvial source. Furthermore, the system’s fluvial sediment is driven by coastal currents over a distance of 1000 km, and thus, is also marine-dispersal-dominated. Therefore, it is a major hybrid dispersal system of distal-to-proximal accumulated and marine-dispersal dominated. Comparing to the characteristics of mud deposition zones and river dispersal systems in the world^1,2^, the Z-M-TSMD’s hybrid nature makes it unique in the world.

## Methods

### Shipboard monitoring and sampling

On Feb. 17, 2014 off the mouth of Oujiang River at Sta. M1 (121.5**°**E, 27.8**°**N) (Fig. 1a) in water depth of 22.5 m R/V Ocean Researcher 5 (OR-5) deployed a mooring (Fig. 1b). It was configured with (in descending order) a downward looking ADCP (Aquadopp), non-sequential sediment trap^51,52^, a laser sediment analyzer (LISST-100X), and a CTD with a turbidity sensor (XR-420). Afterwards, OR-5 took 3 gravity and 7 box cores and sediment samples from the seabed along the mud belt (Fig. 1a) from Feb. 18–22. The ADCP had 1-m bin size and sampling interval of 30 min. LISST-100X had burst intervals of 30 min, having 60 samples per burst with sampling interval of 5 sec. XR-420 had sampling intervals of 30 min. On the same day (Feb. 17), a chartered fishing boat by Nanjing and Tongji Universities also deployed a WatchKeeper buoy (by AXYS, Canada) to measure waves and the weather. Wind speed and direction were collected 2 m above the sea surface at 1-hr intervals with a sampling rate of 1 Hz. Wave parameters including significant wave height, wave period, and propagation direction were sampled at 4 Hz. The chartered fishing boat also conducted hydrographic profiling along a shore-normal transects near Sta. M1 on Feb. 27 and March 9.

On March 13 R/V Ocean Researcher 1 (OR-1) arrived at Sta. M1 to conduct 24-hr hydrographic profiling, water and seafloor sediment sampling before retrieving the sediment trap mooring. The profiling included hourly salinity, temperature, chlorophyll-a, and volume concentration of suspended particles of thirty-two grain sizes (using LISST-100X). Water samples were taken at the surface, mid-depth, and near seabed for the analysis of suspended sediment concentration (SSC) at 2-hr intervals using the method described before^44^. Seabed sediment samples were analyzed for grain-size distribution using the methods described before^44^.

### SSC measurement

On board RV OR-1 water samples were taken at 3-m below the surface (surface), the location of the thermocline (mid-depth) at the time of sampling, and near bed (bottom-instantaneous depth minus 10%) using 10-l X-Niskin bottles mounted on the CTD rosette. Water samples were filtered onboard immediately using a nested filtration system called Catnet, which contained three funnel-shaped Nitex nets stacked together inside a polycarbonate container having the volume of 60 l. The mash sizes of the nets were 10, 63 and 153 μm, respectively. Particles captured on the Nitex nets were carefully washed into a 100 ml PE bottle by hand using deionized distilled water (DDW), which was filtered using GF/F (0.7 μm) filter paper. Great care was taken in the washing process to avoid breakage of floc particles^53^. One liter of the residual water in the Catnet container was later filtered through the GF/F (0.7 μm) filter paper. The filtration separated suspended particles into four different grain-size classes (>153, 63–153, 10–63, and <10 μm). All filter papers were first stored in a refrigerator at −4°C, than dried in the oven at 55 °C in the laboratory for 24 hours and weighed to calculate SSC^43^.

### Conversion from acoustic backscattering to SSC

The active sonar equation has been widely used to convert SSC from backscattering strength for decades^54–56^:

1 $${S}_{v}=2\alpha R+{K}_{c}(E-{E}_{r})+10\,log10[({T}_{T}\,\ast \,{R}^{2})/(L{P}_{T})]+C$$

where S_v_ stands for volume backscattering strength (dB), α is the attenuation coefficient (dB/m), K_c_ is a scale factor (dB/count), E is echo strength (in count), E_r_ is received noise (in counts), T_T_ denotes the transducer temperature (°C), R is the distance along the central beam axis (m), L is transmit pulse length (m), P_T_ is the transmit power (W), and C is a constant (dB). After combining other constants (e.g. T_T_ and E_r,_ etc.) with C, (1) becomes as:

2 $$10\,log10(SSC)=10\,log10[{R}^{2}]+2{\alpha }_{w}R+{K}_{c}E+{C}_{k}$$

where α_w_ is water attenuation calculated by *in-situ* temperature, salinity, and pressure^57^. C_k_ is the combining constant. Both C_k_ and K_c_ are determined by linear regression of backscatter intensity to SSC (mg/l) measured from collected water samples at corresponding depths.

### Static Stability (E)

E is expressed as:

3 $${\rm{E}}=\frac{-1}{{\rho }_{0}}(\frac{\partial {\rho }_{w}}{\partial h})$$

where h is the water depth (m),is the seawater density (g/cm^3^) and is the water density at a reference depth.

### Clay mineral analysis

The analysis was conducted at the Institute of Oceanography, Chinese Academy of Sciences using X-ray Diffraction (XRD) with a D8 ADVANCE diffractometer with CuKα radiation (40 kV and 40 mA) following established procedures^58^.

### Radioisotope analysis

The analyses of ^7^Be and ^210^Pb_ex_ were carried out in the Institute of Earth Sciences, Academia Sinica following the established procedures^26^.

### Grain-size analysis

Grain-size distribution of samples from the sediment trap and seabed were analyzed in the Department of Oceanography, National Sun Yat-sen University following established procedures^59^.

## Electronic supplementary material

Supplementary Information
