# Supplementary material for: A comprehensive sediment dynamics study of a major mud belt system on the inner shelf along an energetic coast
Source: Sci Rep. 2018 Mar 9;8:4229. doi: 10.1038/s41598-018-22696-w (PMC5844877; doi:10.1038/s41598-018-22696-w)
Supplement: Supplementary file 1 — Supplementary Information [file 41598_2018_22696_MOESM1_ESM.pdf]

Supplemental Information

A comprehensive sediment dynamics study of a major mud belt system on the inner shelf along an energetic coast

James T. Liu[1], Ray T. Hsu[1], Rick J. Yang[1], Ya Ping Wang[2], Hui Wu[2], Xiaoqin Du[1,3], Anchun Li[4], Steven C. Chien[1], Jay Lee[1,5], Shouye Yang[6], Jianrong Zhu[2], Chih-Chieh Su[7], Yi Chang[8], Chih-An Huh[9]

[1]Department of Oceanography, National Sun Yat-sen University, Kaohsiung, Taiwan, ROC

[2]State Key Laboratory of Estuarine and Coastal Research, East China Normal University, Shanghai, China

[3]School of Ocean Science and Technology, Zhejiang Ocean University, Zhoushan, Zhejiang, China

[4]Institute of Oceanology, Chinese Academy of Science, Qingdao, China

[5]Taiwan Ocean Research Institute, National Applied Laboratories, Kaohsiung, Taiwan, ROC

[6]State Kay Laboratory of Marine Geology, Tongji University, Shanghai, China

[7]Institute of Oceanography, National Taiwan University, Taipei, Taiwan, ROC

[8]Department of Hydraulic and Ocean Engineering and Institute of Ocean Technology and Marine Affairs, National Cheng-Kung University, Tainan, Taiwan, ROC

[9]Institute of Earth Sciences, Academia Sinica, Taipei, Taiwan, ROC

Vertical structures of the flow field and cumulative sediment transport in the lower water column 11 m above seabed

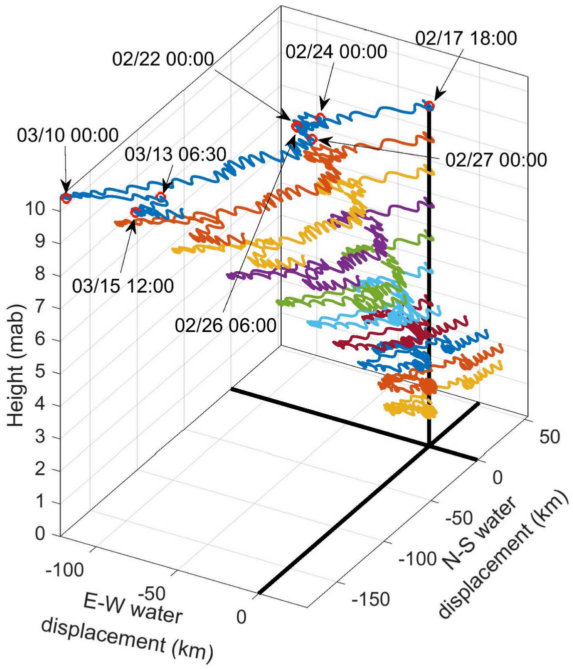

Figure S1. Water displacement in the lower water column. Progressive vector plot at 1-m intervals of the current measured by the ADCP on the mooring between 1 and 11 mab (meter above the bed). The circles indicate time of weather-related flow reversals.

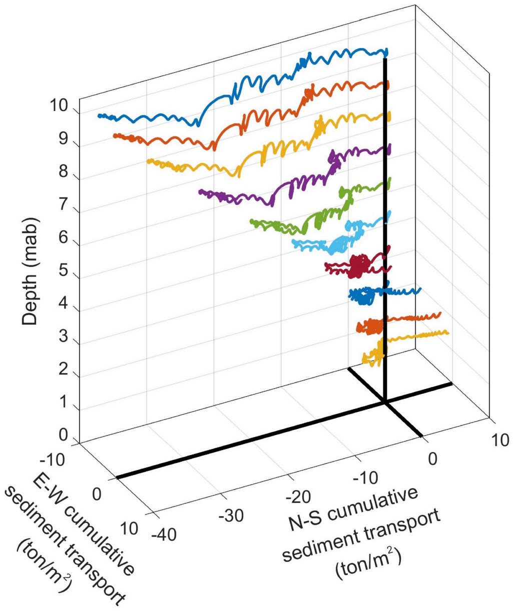

Figure S2. Cumulative sediment transport in the lower water column. Progressive vector plot of sediment transport based on the product of the measured flow and converted SSC from the echo intensity of the ADCP on the mooring at 1-m intervals between 1 and 11 mab.

Quantification of Tidal Influence

The tidal influence is expressed by tidal energy ratio (ER) as follows:

$$\mathrm{ER} = \frac{\sum[v(t_i)-v_p(t_i)]^2}{\sum[v(t_i)-\bar{v}]^2} \times 100\% \qquad (1)$$

in which $v(t_i)$ is a variables in a time series, $v_p(t_i)$ is the tidal part of the time series expressed as:

$$v_p(t) = \sum_{k=1}^{m} a_k \cos(\omega_k t - \theta_k) \qquad (2)$$

in which $\bar{v}$ is mean value of the observed time series; ω_k is radian frequency ($2\pi/T_k$); T_k is period of the kth tidal constituent; α_k and θ_k are amplitude and phase of the kth tidal constituent respectively; m is the number of data points in the time series.

Basically, ER is the ratio between tidal variance and total variance. The higher the ER, the greater the tidal influence. Some variables measured on the mooring at difference depths are tabulated below to illustrate different degrees of tidal influence on variables.

Table S1. Energy Ratio (ER-%) of Tidal Variance/Total Variance of Observed Time Series

Water depth	Sal @ 8	Temp @ 8	Temp @ 14	Along @ 1.5	Across @ 1.5	Along @ 8	Across @ 8	Along @ 10	Across @ 10	SSC @ 1.5	SSC @ 9	SSC @ 11
99.1	15.9	20.0	18.3	44.1	93.1	28.6	96.7	29.1	96.0	29.0	27.4	35.5

Note: The number after the @ sign is the elevation in meters above the bed (mab). 'Along' stands for alongshore velocity, 'Across' stands for across-shore velocity.

The above results indicate that the tide was the primary forcing for the water depth changes. The tide also dominated the flow field in the across-shore flow component (ER over 93%), but the alongshore flow was greatly affected by non-tidal processes (ER from 28.6%-44.1%) that included the Zhe-Min Coastal Current, Changjiang buoyant plume, and other coastal forcing. The tidal influence was week in the SSC fluctuations (ER between 27.4%-35.5%). The increase of ER away from the seabed was probably because of reduced wave influence and increased influence of the tidal regime.

The salinity and temperature were the least affected by the tide (ER smaller than 20%). This is not surprising because both were largely determined by the strength of the Changjiang

fresh water discharge and other current systems such as ZMCC and KBC. Solar heating and cooling, and weather events also affected the water temperature.

Both tide and the wind field exerted influence on the wave field. The tidal influence can be seen in the temporal changes in the increase of the significant wave height (Hsig) that coincided with the spring tide (Fig. S3). The influence of the wind can be seen from the shift of the wave direction from ENE (0-90 deg.) to SE (90-180 deg.) during episodes of southerly winds.

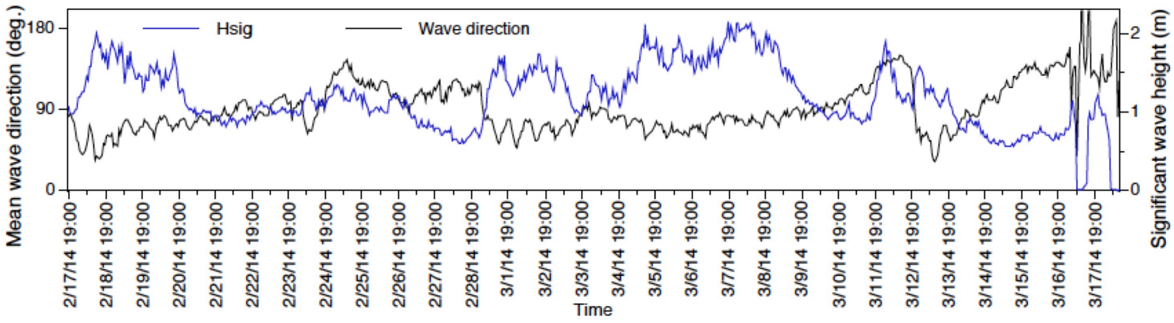

Figure S3. Temporal changes of the wave field. Significant wave height increased during spring tide and wave direction (incident angle) shifted from ENE to SE during episodes of southerly winds.

Measurement of Suspended Sediment Concentrations (SSC)

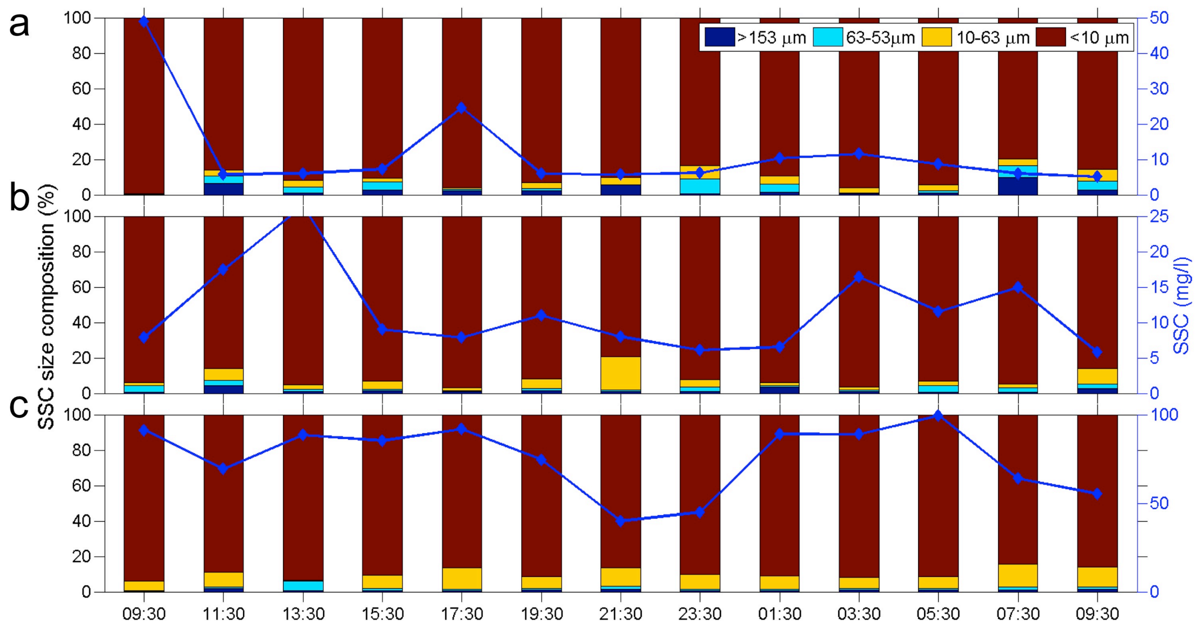

Figure S4. SSC and grain-size composition. The total SSC (blue curve) and the grain-size composition (cumulative % by weight) of measured from water samples taken at (a) surface, (b) mid-depth, and (c) bottom at bi-hourly intervals on March 17-18, 2014.

The weight-composition indicates the fine-grained (finer than medium silt) particles dominated the suspended sediment composition (over 80%) in the water column. The higher presence of coarser particles (greater than 63 μm) at the surface was probably due to the presence of biogenic particles such as phytoplankton. The total SSC (mg/l) was 46.9 mg/l at the surface, 46.2 mg/l at mid-depth, and 298.5 mg/l near the seabed. The total SSC was one order of magnitude greater near the bed than at the surface and mid-depth suggesting that after suspended sediments settled into the lower water column, they are prevented from being mixed upward. Also, the high SSC was contributed from resuspension of seabed sediment.

Numerical Simulation of the Observed Water Column Structures

A numerical model with large domain of the Western North Pacific (Fig. S5a) was run to provide flow, salinity, and temperature in the area of interest (Fig. S5b). The model had 367x319 horizontal grids and sigma coordinate of 31 vertical layers. The model grid resolution varied from several hundred meters inside the Changjiang river mouth to 12 km near the open boundaries.

At the open ocean boundaries, the water fluxes were composed of the tidal flow and ocean currents. The tidal flow consisted of 11 tidal constituents (M_2，S_2，N_2，K_2，K_1，O_1，P_1，Q_1，M_4，M_6，MS_4) based on National Astronomical Observatory Tide (NaoTide) data. Ocean currents were obtained from the monthly Simple Ocean Data Assimilation (SODA) climatological data. The temperature and salinity at the open ocean boundaries were specified with radiation conditions based on SODA climatological data. At the upstream Changjiang open boundary, the water fluxes were based on the hourly river discharge measured at Datong Gauging Sta. The temperature was given by empirical climatic temperature values and salinity was set to zero. At the sea surface boundary, wind data for surface momentum, atmospheric temperature, pressure, humidity, and cloud cover for heat flux at air-sea interface were obtained from the European Center for Medium-Range Weather Forecasts (ECMWF) reanalysis data products with 0.5-degree spatial resolution at 6 hr time intervals. The temperature and salinity for initial conditions were based on SODA climatological data.

The model run started on January 1, 2013, through the entire year of 2014. The model time step was 180s. More details about the numerical model setup and model validation with in situ tide and salinity data can be found in Wu et al. (2011) and Wu et al. (2014).

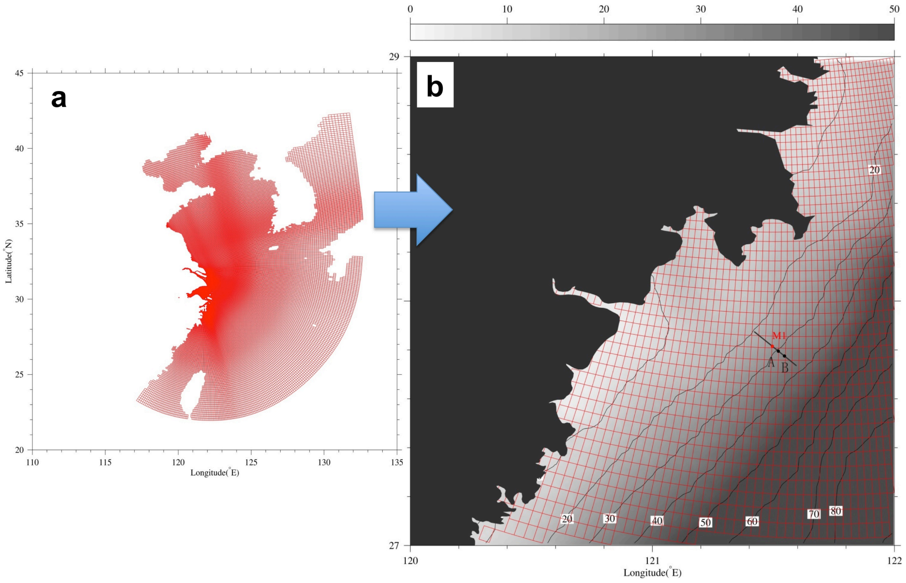

Figure S5. Numerical modeling domain. (**a**) Model grid; (**b**) enlarged local grid in which

deployment Sta. M1 and two checkpoints A, B are shown. Numbers in boxes mark the depths of bathymetric contours. The grid was generated by the software Delft-3D (https://oss.deltares.nl/web/delft3d, version 4.01) using bathymetric data from the ETOPO2 (https://www.ngdc.noaa.gov/mgg/global/etopo2.html) dataset. The figure was plotted with the software Gri (http://gri.sourceforge.net/gridoc/html/index.html) version 2.12.7.

References:

Wu, H.; Zhu, J., Shen, J., and Wang, H.， 2011. Tidal modulation on the Changjiang River plume in summer. J. Geophys. Res., Vol. 116, C08017, doi:10.1029/2011JC007209

Wu, H., Shen, J., Zhu, J., Zhang, J., and Li, L., 2014. Characteristics of the Changjiang plume and its extension along the Jiangsu Coast. Cont. Shelf Res., 76,102-123.

Empirical Orthogonal/Eigen Function Analysis of Sediment Trap Samples

Sinking particles accumulated in sediment trap for about 15-cm in height (Fig. S6a). The trap sediment was sampled at 1 cm intervals for various analyses. The entire trap sediment looked homogenous having grayish brown color with some horizontal coloration, no apparent internal structures were visible to the naked eye. However, the x-ray radiograph shows alternating dark-light laminations, whose appearance could be related to the spring tide, which coincided with larger mean grain-sizes (Fig. S6b). The water content (37-55%) and mean grain-size (9-16 μm) trends show mirror images (Fig. S6a, b), which is common in sediment cores. The cumulative percent (by weight) shows a relative composition of about 20% clay and 80% silt. Only a minute amount of sand appeared in the trap and is not visible in the cumulative plot. The TOC content in the trap ranged from 0.5-1.1, most of which were contributed by marine-sourced particles having C/N ratio from 7.4-10 (Fig. S6d). However, $^{13}\delta$C-based terrestrial fraction (F_t) suggests about equal contributions from land and sea (Fig. S6e). Percentages of 7 minerals including mica (ilite), kaolinite, chlorite, quartz, k-feldspar, plagioclase, and calcite are shown in Figure S6f.

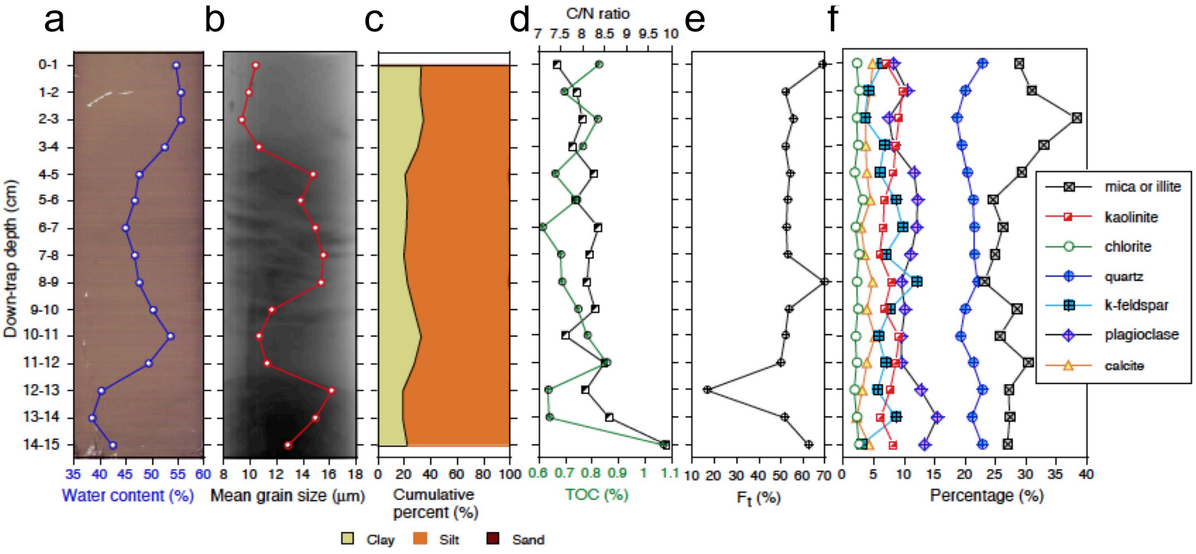

Figure S6. Characteristics and properties of sinking particles in the sediment trap. (**a**) Water content of the particles in the trap plotted over the photo of the trap material; (**b**) Mean grain-size plotted over x-ray radiograph of the trap material; (**c**) Cumulative percentage of clay, silt, and sand; (**d**) TOC content and C/N ratio, (**e**) Terrestrial fraction (F_t); (**f**) Percentages of mica (ilite), kaolinite, chlorite, quartz, k-feldspar, plagioclase, and calcite.

Because of the multivariate nature of the measured variables of the particles in the sediment trap, Empirical Orthogonal/Eigen Function (EOF) technique was used to extract

information from the co-variability of the variables. The variables were arranged in the following order with different implications: the percentages of clay, silt, sand represent physical properties of the sediment grains; total ^{210}Pb (not shown), TN (not shown), TOC, C/N, F_t represent terrestrial factors; three clay minerals ilite, chlorite, and kaolinite are fluvial source indicators; quartz, k-feldspar, and plagioclase are plutonic rock indicators; and calcite could either be biogenic or terrestrial indicator.

The result shows that the first mode explains 43.4% of the correlations (normalized co-variance). The 14 variables were grouped into two groups according to sign of the eigenvector. This mode distinguished clay (marked red, in the minus-sign group) from silt and sand (marked blue, in the plus-sign group), which indicates the effect of hydrodynamic sorting in the particle transport process that separated the fines (clay) from the coarse fraction (silt and clay). Co-varied with clay are organics, F_t, the three clay minerals, and calcite (Fig. S7a). This mode reveals the fundamental nature of the sinking particles that the clay-sized particles were of terrestrial origin, and they were associated with organics, clay minerals, and terrestrial calcite.

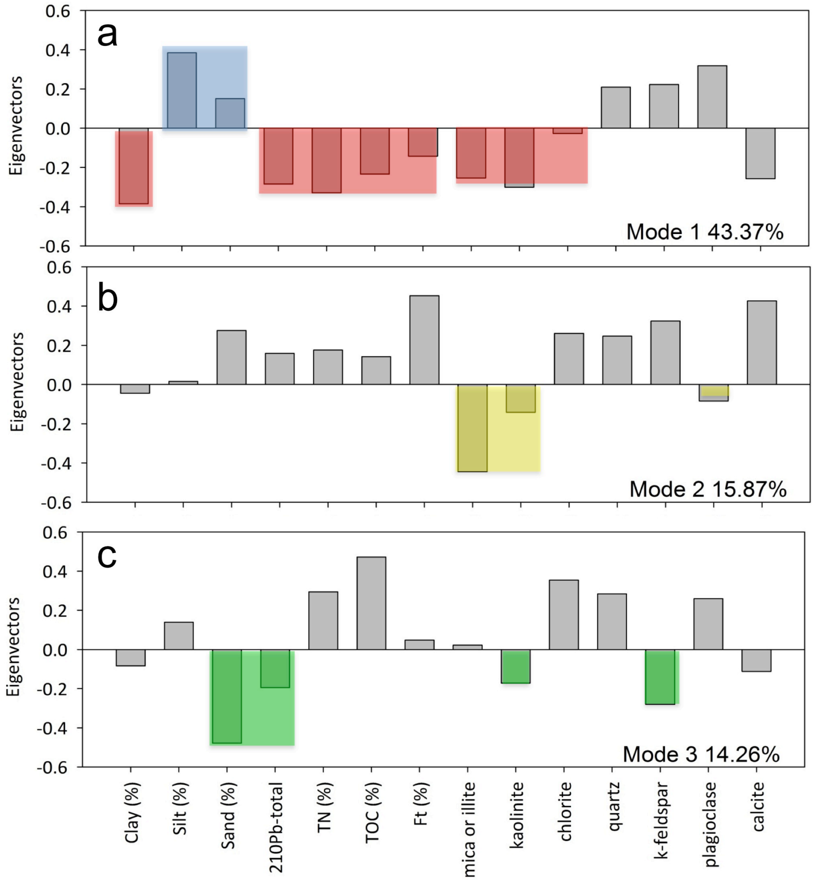

Figure S7. Eigenvector plots of the first three modes. The variables were grouped according the sign of their eigenvectors. (**a**) The fist mode explains 43.4% of the co-variability; (**b**) The second mode explains 15.9%; (**c**) The third mode explains 14.3%.

The second mode sets mica (ilite), kaolinite, and plagioclase apart from other variables (Fig. S7a). This mode points to the provenance contrast of weathering product of granite (marked in yellow) vs. non-granitic sources. In the third mode clastic indicators of sand, total ^{210}Pb, kaolinite, k-feldspar, and calcite (marked in green) are in the negative group and the organics, F_t and silt were in the positive group. This mode might show provenance contrast between marine-sourced coarse clastic provenance vs. silty organics of terrestrial origin. These modes suggest that physical processes that transported the suspended sediment had the greatest influence on the properties of the sinking particles and the source-signals they carry. The different provenance in the region and the marine environment also influence the particle properties in the secondary and tertiary degrees.

TS Mud Belt and Locations of Cores G3 and ZS-3 on the CYR

Contours of the mean grain-sizes are extracted from Huh et al. (2011) and were plotted over the bathymetric contours in the TS to show the location of the TS mud belt. Both Cores G3 and ZS-2 were located on the CYR. G3 was located in water depth of 26.3 m, outside the TS mud belt and ZS-2 was located in water depth of 25 m just inside the boundary of the TS mud belt and a little closer to the mouth of Zhuoshui River (Fig S8a). The distance between the two coring sites was 16.9 km and ZS-2 was closer to the Zhuoshui River (Fig. S8b).

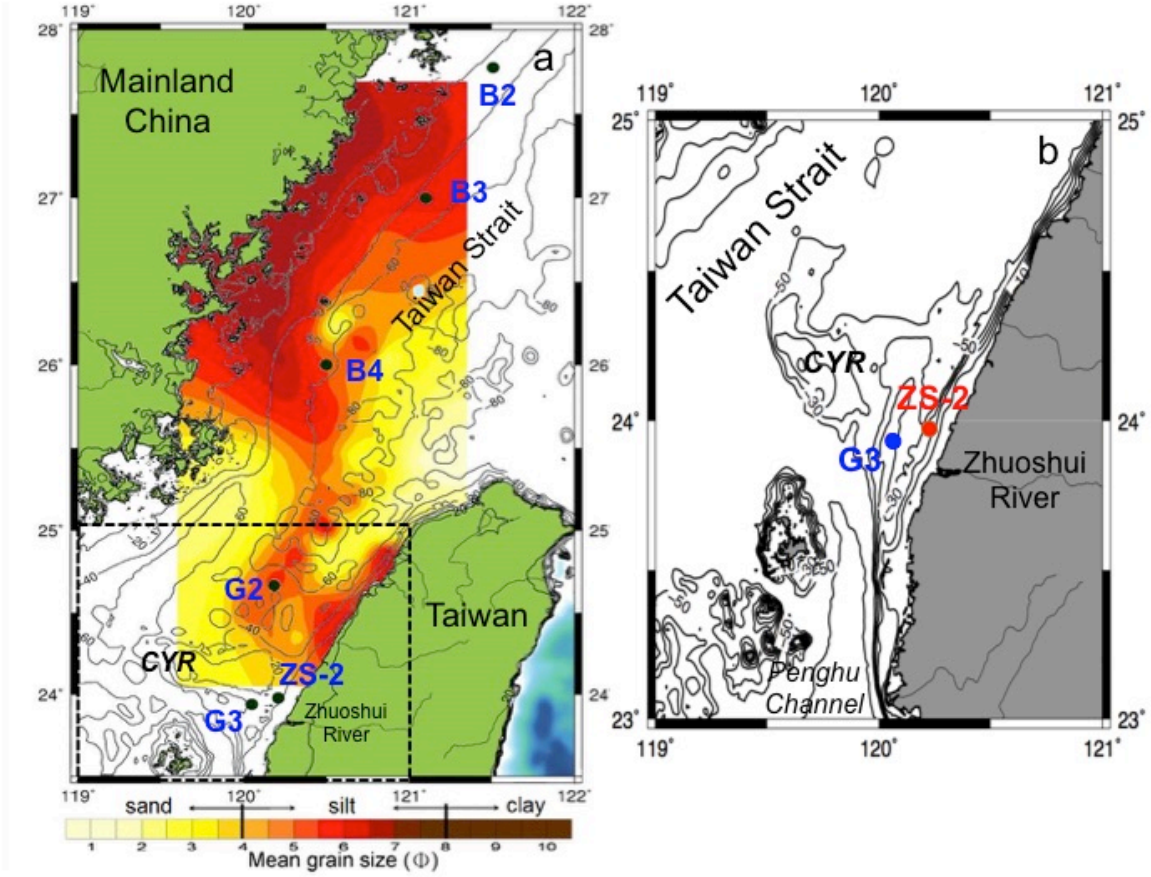

Figure S8. Locations of Cores G3 and ZS-2 and the Taiwan mud belt. (**a**) Contours of mean grain-sizes[26] on phi scale are plotted over the bathymetric contours of TS. The Taiwan mud belt originates on the west coast of Taiwan, extends westward into the TS and northeastward, eventually joins the mud belt on Zhe-Min Coast. The Taiwan mud belt is located immediately north of CYR. The dashed box is an enlarged map (**b**) showing locations of G3 and ZS-2 and CYR. G3 is outside the mud belt and ZS-2 is within the boundary of the mud belt off the mouth of Zhuoshui River. ZS-2 was taken on R/V OR-3 in summer 2015 for a different study. The bathymetric contours with coring sites in (**a**) were plotted by GMT 5.1.2 software (http://gmt.soest.hawaii.edu) using data from Taiwan's Ocean Data Bank

(http:/www.odb.ntu.edu.tw) with the grid resolution of 200 meters. The map then was merged with the grain-size contours[26] in PowerPoint and geographic names were added. The base bathymetric map in (**b**) was plotted by GMT 5.1.2 software using data from Taiwan's Ocean Data Bank with the grid resolution of 200 meters, which was later imported into PowerPoint. Coring sites and geographic names were later added in PowerPoint.

Long Gravity Cores along the Mud Belt

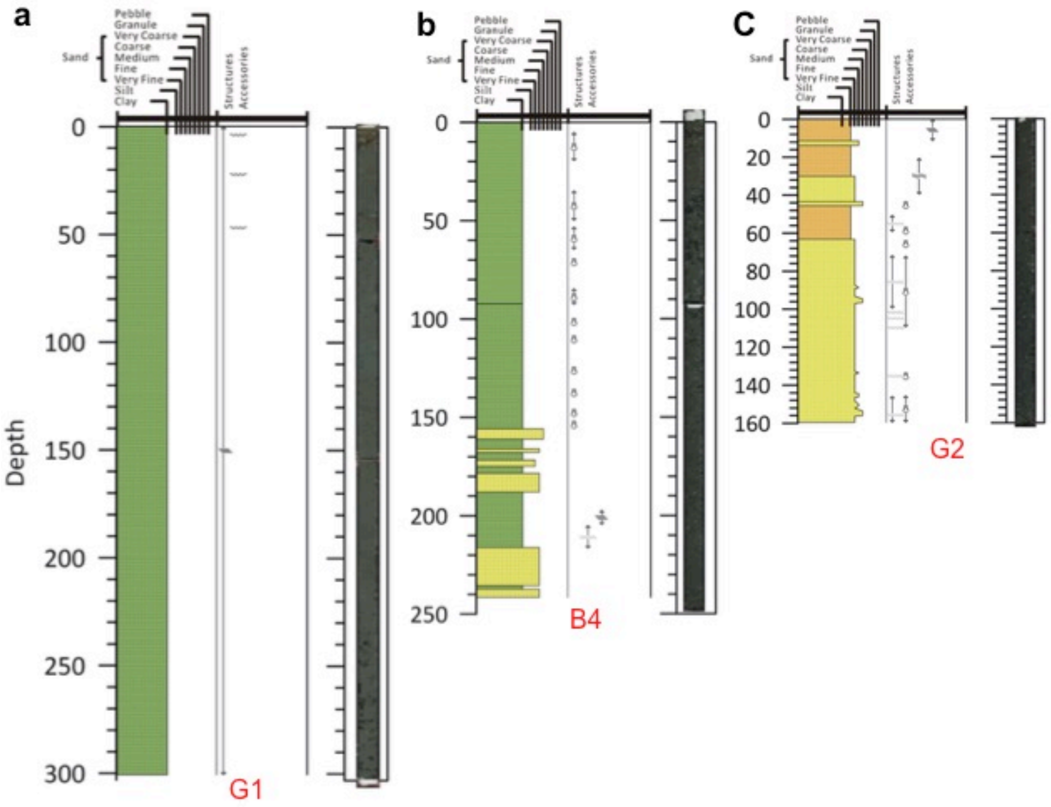

Figure S9. Lithology and photos of the gravity cores G1, B4, and G2. These cores show decreasing thickness of clay from the mouth of Changjiang R. to the mud belt in the TS. Conversely, the type of internal sedimentary structures increases and the frequency and thickness sand layers also increase.

The clay thickness was over 3 m at G1, about 1.6 m at B4 (from core top to the first appearance of the sand layer), and the thickness of silt was about 0.7 m at G2 with inter-bedded sand layers. The overall core attributes suggest that the fine-grained (clay) sedimentary process was the strongest at the northern end of the mud belt and the physical processes dominated the sedimentation. At the mid-point of B4, biological processes became important of the sedimentary process and the supply of clay from Changjiang R. probably reduced. At G2, which is at the junction between the Zhe-Min mud belt and the TS mud belt, the dominant sedimentary features were silt deposition in which physical and biological processes were both important.

Boundaries of Current Systems Delineated by Sea Surface Temperature Gradients

Composites of multi-year sea surface temperature were acquired by AVHRR (Advanced Very High Resolution Radiometer) on NASA's satellites (https://podaac.jpl.nasa.gov/AVHRR-Pathfinder). Entropy-based edge detection method was than used to obtain gradient magnitude of frontal pixels (Chang and Cornillon, 2015; Shimada et al., 2005). Areas of high-density gradient delineate the boundaries/fronts between different water masses driven by different ocean current systems. The front between ZMCC and the ambient ECS and TS (the black line marked by circled number 1) is better developed in winter (Fig. S10), which was the best developed in February (Fig. S10b). This black line forms a large bend in northern TS, whose apex (marked by the orange arrow) is the position where Taiwan-derived mud belt joins the mud belt along the Zhe-Min Coast. The other frontal system marked by circled number 2 is the KBC, which is also best developed in February (Fig. S10b).

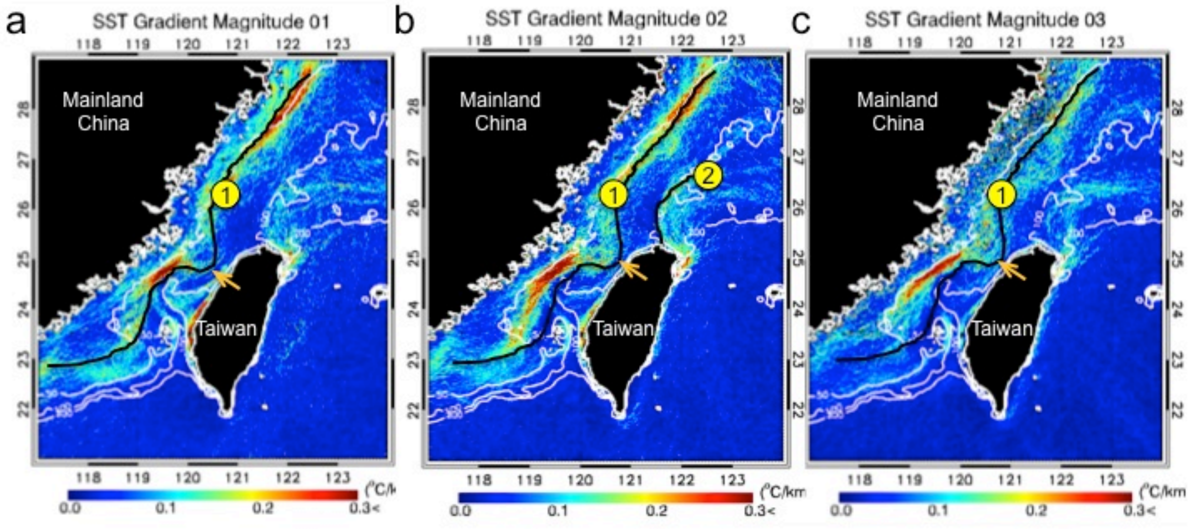

Figure S10. Sea-surface temperature gradients delineating boundaries of current systems. Satellite-based sea surface temperature gradients for the months of (**a**) January; (**b**) February; and (**c**) March. The circled number 1 indicates the boundary of ZMCC, and number 2 indicates that of KBC. The arrow points to where Taiwan-sourced mud belt joins the mud belt on the Zhe-Min Coast. The maps were generated by IDL (Interactive Data Language, version 8.1) software (http://www.harrisgeospatial.com/ProductsandTechnology/Software/IDL.aspx).

References

Chang, Y. and Cornillon, P., 2015. A comparison of satellite-derived sea surface temperature

fronts using two edge detection algorithms. Deep Sea Research Part II: Topical Studies in Oceanography, 119: 40-47.

Shimada, T., Sakaida, F., Kawamura, H. and Okumura, T., 2005. Application of an edge detection method to satellite images for distinguishing sea surface temperature fronts near the Japanese coast. Remote sensing of environment, 98(1): 21-34.
